# Enhanced Luminous Transmission and Solar Modulation in Thermochromic VO_2_ Aerogel-like Films via Remote Plasma Deposition

**DOI:** 10.1021/acsami.5c07264

**Published:** 2025-09-22

**Authors:** Jose Manuel Obrero, Gloria Patricia Moreno-Martinez, Teresa Cristina Rojas, Francisco Javier Ferrer, Francisco G. Moscoso, Lidia Contreras-Bernal, Javier Castillo-Seoane, Fernando Nuñez-Galvez, Francisco Javier Aparicio Rebollo, Ana Borras, Juan Ramon Sanchez-Valencia, Angel Barranco

**Affiliations:** † Nanotechnology on Surfaces and Plasma Laboratory, Materials Science Institute of Seville (CSIC-US), C/Américo Vespucio 49, 41092 Seville, Spain; ‡ Tribology and Surface Protection Group, Materials Science Institute of Seville (CSIC-US), C/Américo Vespucio 49, 41092 Seville, Spain; § 203374 Centro Nacional de Aceleradores (Universidad de Sevilla, CSIC, Junta de Andalucía), Avda. Tomas Alba Edison 7, 4092 Sevilla, Spain; ∥ Departamento de Física Atómica, Molecular y Nuclear, Universidad de Sevilla, Aptdo 1065, 41012 Sevilla, Spain; ⊥ Center for Nanoscience and Sustainable Technologies (CNATS), Departamento de Sistemas Físicos, Químicos y Naturales, 16772 Universidad Pablo de Olavide, Ctra. Utrera km. 1, Sevilla 41013, Spain; # Departamento de Ingeniería y Ciencia de los Materiales y el Transporte, EPS-Universidad de Sevilla, c/Virgen de África 7, 41011 Sevilla, Spain

**Keywords:** aerogel-like films, VO_2_, thermochromic, remote plasma synthesis, smart windows, optical films

## Abstract

Vanadium dioxide (VO_2_) is a thermochromic material that undergoes a phase transition from a monoclinic semiconducting state to a rutile metallic state at 68 °C, a temperature close to room temperature. This property makes VO_2_ particularly valuable in applications such as optical and electrical switches, data storage, neuromorphic computing, and remarkably dynamic smart windows for solar radiation control. VO_2_ typically needs to be synthesized for these applications as nanostructured thin films. Over the past few decades, significant efforts have been made to control the thermochromic properties of VO_2_ through crystal structure tuning, doping, and the development of VO_2_ nanocomposites. Additionally, introducing nano- and mesoporosity has been shown to enhance the optical properties of thermochromic VO_2_ films. This study presents a methodology for producing highly porous, aerogel-like V_2_O_5_ films, which can be thermally processed to form aerogel-like VO_2_ films. This process is based on sequential plasma polymerization and plasma etching to produce aerogel-like V_2_O_5_ films that are annealed to yield ultraporous nanocrystalline VO_2_ films. The sacrificial vanadium-containing plasma polymers are obtained by remote plasma-assisted vacuum deposition (RPAVD) using vanadyl porphyrin as a precursor and Ar as plasma gas. Additional reference compact films VO_2_ films are obtained by a direct RPAVD process using the same precursor and oxygen plasmas in combination with thermal annealing. The aerogel-like VO_2_ films show exceptional thermochromic performance with luminous transmittances higher than 54%, solar modulation up to 18.8%, and IR modulation up to 35.5%. The presented plasma methodology is versatile, allowing both the synthesis of VO_2_ plasmonic structures to enhance the thermochromic response and the encapsulation of films to improve their stability in air dramatically. Additionally, this solvent-free synthetic method is fully compatible with doping procedures, scalable, and holds great potential for designing and optimizing smart window coatings.

## Graphical abstract

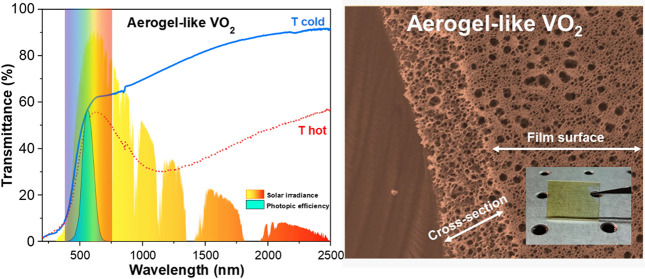

## 1 Introduction

The escalating demand for high energy, driven primarily by innovative technologies, has led to a significant increase in carbon dioxide emissions, directly contributing to global warming. This demand constitutes approximately 30–40% of the overall energy consumption in conventional households.^1^ To mitigate this surge, developing materials capable of reducing environmental impact while enhancing indoor comfort, particularly concerning heating/cooling, ventilation, and lighting, has become imperative. In this context, materials exhibiting thermochromic properties present a promising solution. These materials can transmit the entire solar spectrum at low temperatures and selectively reflect infrared radiation at higher temperatures. In recent decades, vanadium­(IV) oxide has emerged as a promising candidate for smart windows due to its reversible metal–insulator transition (MIT) at a relatively low transition temperature (*T*_t_) of 68 °C (341 K) for bulk material.^2^ Below this threshold, VO_2_ maintains a monoclinic lattice (*P*2_1_/*c*, VO_2_(M)) that switches to rutile (*P*4_2_/*mnm*, VO_2_(R)) above this temperature.^3^ This phase transition is accompanied by a substantial change in the optical and electrical properties, which are reversible by cooling.^3,4^ The VO_2_(M) phase is semiconducting and transparent to infrared solar radiation, while VO_2_(R) is metallic and reflects solar radiation. The VO_2_ metal–insulator transition arises from the alternating displacement of vanadium atoms, which changes the V–V distances and the interaction between vanadium d orbitals and oxygen orbitals, thereby shifting the Fermi level.^4,5^

In the field of smart windows, key limitations hinder the performance of thermochromic VO_2_-based materials. For optimal thermochromic behavior, the main requirements include an appropriate transition temperature (*T*_t_), high luminous transmittance (*T*_lum_), a significant modulation of solar transmittance (Δ*T*_sol_), and good stability against oxidation.^1^ To effectively implement VO_2_ coatings on conventional glass for smart window applications, the material must fulfill the following criteria: (i) Tt close to ambient temperature (∼25 °C);^6,7^ (ii) *T*_lum_ > 60%;^8–10^ (iii) Δ*T*_sol_ > 10%;^11–13^ (iv) excellent near-infrared (NIR) transmittance modulation capacity (Δ*T*_IR_) > 10% ^14,15^ and (v) long-term stability.^16^ Numerous strategies have been explored to lower the transition temperature while improving the thermochromic and solar energy efficiency of a VO_2_ film.^17^ However, the improvement of Δ*T*_sol_ typically occurs at the expense of *T*_lum_, which implies a complex challenge when optimizing the synthesis of these films. The film microstructure modification appears to be the key to addressing this challenge. In recent years, a certain balance between Δ*T*_sol_ and *T*_lum_ has been found to improve thermochromic efficiency by modifying part of the VO_2_ structure, such as crystal morphology,^18^ particle size,^19^ and porosity.^20–22^ Other common strategies to decrease *T*_t_ include doping and codoping the VO_2_ with different elements.^9,23–25^ However, balancing the transition temperature with optical properties poses an even more significant challenge. Nonetheless, previous studies have demonstrated that, in comparison to other approaches, introducing increased porosity as a secondary component into VO_2_ films offers a viable alternative for enhancing Δ*T*_sol_ without compromising *T*_lum_ and Δ*T*_IR_,^17,21,26^ being this strategy also beneficial for lowering the phase transition temperature.^27^

We have recently reported a cyclic plasma-based process that enables the synthesis of aerogel-like, conformal TiO_2_ thin films with low density and ultrahigh porosity values characteristic of aerogel materials.^28^ In this work, we extend this approach to fabricating aerogel-like thermochromic VO_2_ films. Thus, the aerogel-like VO_2_ film deposition combines the plasma polymerization by remote plasma-assisted vacuum deposition (RPAVD)^29–33^ of a V-containing precursor and remote soft plasma etching (SPE), followed by a reduction thermal treatment to get the appropriate phase and stoichiometry. Our research delves into the impact of film thickness and porosity on their thermochromic properties, including *T*_lum_, Δ*T*_sol_, and Δ*T*_IR_, as well as their transition temperature. We have also explored the encapsulation of the films with a transparent plasma polymer to preserve the stability of the VO_2_ aerogel-like structure in the face of oxidation. This work presents the fundamental principles of this innovative method for producing robust and high-performance thermochromic VO_2_-based coatings suitable for smart window applications by industrially scalable procedures.

## 2 Experimental Section

### 2.1 Fabrication of Aerogel-like VO_2_

The experimental methodology and setup are fully described in ref 28, applied to the synthesis of aerogel-like TiO_2_, and it consists of a cyclic procedure combining remote plasma polymerization (RPAVD) and plasma etching (SPE) to yield porous oxide films. This combined procedure is repeated several times to increase the thickness of the films. The films were deposited on pieces of Si (100) and fused silica slides. The characteristics of each synthetic step are the following:

(a) **Deposition of V-containing plasma polymer**: The precursor Vanadyl phthalocyanine (VOPc, dye content >90%) from Sigma-Aldrich was used as received. VOPc plasma polymers were deposited by remote plasma-assisted vacuum deposition (RPAVD). The details of the RPAVD process and a complete description of the experimental setup is reported elsewhere.^30,31^ In resume, the plasma polymerization was carried out in an electron cyclotron resonance (ECR) 2.45 GHz microwave (MW) plasma reactor, with the sample holder 9 cm downstream from the Ar discharge, with the samples facing down. The precursor was sublimated from a Knudsen cell facing the samples at ∼9 cm from the holder position under an Ar plasma of 150 W at 10^–2^ mbar. Before deposition, the system was evacuated to a base pressure of 10^–6^ mbar. Argon (Ar) gas was introduced using a calibrated mass flow controller. The deposition rate of the growing film was monitored with a quartz crystal microbalance (QCM) positioned near the sample holder. Under these conditions, deposition was performed at room temperature, as measured by an encapsulated thermocouple connected to the sample holder. The VOPc RPAVD plasma polymers were used as sacrificial layers for the synthesis of ultraporous oxides by plasma etching.
(b) **Remote soft plasma etching (SPE-O**_**2**_ **)**: Plasma etching treatment of the VOPc films was carried out in the same reactor, rotating the sample holder to face the plasma, reducing the distance to the plasma by 4 cm, and heating the sample holder to 150 °C. O_2_/Ar gas (1:1) was introduced as plasma gas. The treatment was carried out at 10^–3^ mbar with a plasma power of 300 W. The treatment duration was 60 min. These conditions were selected to ensure complete polymer oxidation and formation of ultraporous V_2_O_5_ films.
(c) **Development of V**_**2**_ **O**_**5**_ **ultraporous films with controlled thickness**: The RPAVD and SPE steps were repeated several times to control the thickness of the ultraporous V_2_O_5_ film. Hereafter, we name the combination of these two steps a synthetic cycle C*_n_*, being *n* the number of repetitions. This cyclic procedure allowed us to incrementally increase the thickness of the oxide films up to several hundreds of nanometers, as shown in the following sections.
(d) **Fabrication of reference compact V**_**2**_ **O**_**5**_ **films**: A set of V_2_O_5_ referred hereafter as compact reference films, were deposited using a modified procedure using the same experimental setup and samples location in the reactor by sublimating VOPc in the presence of a remote reactive remote O_2_ plasma (RPAVD-O_2_). The experimental conditions were a pressure of 10^–3^ mbar and 300 W of MW power. The RPAVD-O_2_ was carried out at room temperature. These deposition conditions result in yellowish V_2_O_5_ films. A 10 min after-deposition O_2_ plasma treatment (SPE) was applied at the same MW power and pressure to ensure complete oxidation of the sample surfaces.
(e) **Synthesis of VO**_**2**_ **films by thermal annealing**: The V_2_O_5_ films produced using the previously described synthetic procedures were thermally annealed to yield stoichiometric VO_2_ films. The thermal process was carried out in an atmosphere-controlled furnace in an argon environment (70 sccm Ar flow) using a EUROTHERM 2408 temperature controller. A heating ramp of 5 °C·min^–1^ was applied. This annealing process was carried out for 30 min at 480 °C to achieve the maximum conversion of V­(V) to V­(IV) (see next sections). For aerogel-like samples with thicknesses below ∼200 nm, the annealing process was shortened to 5 min. Afterward, the film was left to cool down back to room temperature.
(f) **Thin film encapsulation by Adamantane remote plasma polymers**: Adamantane plasma polymers were deposited by RPAVD to encapsulate selected porous VO_2_ samples in the same plasma reactor. A complete description of this deposition procedure can be found elsewhere.^31,33^ In resume, adamantane powder (≥99%, Sigma-Aldrich) was sublimated inside the chamber from a heated container at 40 °C in the presence of an Ar plasma at 2·10^–2^ mbar and 150 W. The deposition was carried out at room temperature.

### 2.2 Characterization Methods

High-resolution field emission scanning electron microscopy (FESEM) images of the samples deposited on silicon wafers were obtained in a Hitachi S4800 field emission microscope, working at 2 kV. Cross-sectional views were obtained by cleaving the Si (100) substrates. Scanning transmission electron microscopy (STEM), high-resolution transmission electron microscopy (HRTEM), and high-angle annular dark field (HAADF)-STEM images were acquired in a Tecnai G2 F30 S-Twin STEM from FEI, equipped with a HAADF detector from Fischione with a 0.16 nm point resolution. For this, portions of each sample type were mechanically scraped and placed in the microscopy grids. Electron energy loss spectroscopy (EELS) was performed using a QUAMTUM Gatan Imaging Filter (GIF) attached to the microscope. The Electron energy loss spectra (EELS) were collected in Diffraction mode using a camera length of 560 mm with a spectrometer collection angle of 0.7 mrad. To minimize electron beam irradiation and avoid sample reduction, a smaller condenser aperture and a reduced probe size were employed, resulting in a lower current and electron dose.^34,35^ This approach was necessary as vanadium oxides are prone to reduction under high electron beam doses. Under these conditions, the energy resolution system was ∼1 eV. After experimental acquisition, the data were processed using the Gatan Digital Micrograph software. Selected area electron diffraction patterns (SAED) were also recorded with low electron dose. V_2_O_5_ and vanadium­(IV) acetyl-acetonate powders from Aldrich were used as reference samples of V^5+^ and V^4+^ for the EELS characterization of the samples. These EELS patterns of the references are the same as those reported in the bibliography for V_2_O_5_ and VO_2_.^34,36^

X-ray diffraction at a grazing incidence (GIXRD) using Cu Kα (50 kV, 1 mM) analysis was carried out on a Bruker D8 Discover diffractometer coupled to a 2D detector (Eiger 2R 500 K), with an angle of incidence set at 0.5°. The measurement span ranged from 10 to 60°, with increments of 0.02° and a time step of 90 min.

Variable angle spectroscopic ellipsometry (VASE) was acquired in a Woollam V-VASE ellipsometer. The optical constants were modeled by fitting the spectra to the Cauchy model.

Rutherford Backscattering Spectroscopy (RBS) and Nuclear Reaction Analysis (NRA) characterizations were performed at the 3 MV Tandem Accelerator of the National Centre of Accelerators (Seville, Spain). RBS measurements were performed with α-particles of 2.0 MeV and a passivated implanted planar silicon (PIPS) detector set at a 165° scattering angle. NRA was used to determine C, N, and O elements in the films from the ^12^C­(d,p)^13^C, ^14^N­(d,α_1_)^12^C y ^16^O­(d,p^1^)^17^O nuclear reactions using deuterons of 1.0, 1.4 y 0.9 MeV, respectively. The spectra were obtained using a particle detector set at 150° collection angle and a 13 μm thick Mylar filter to stop the backscattered particles. NRA and RBS spectra were simulated and fitted using the SIMNRA 6.0 code.^37^ The film densities were determined from the combined RBS and NRA analyses of the content per square centimeter and thickness values obtained from cross-sectional FESEM micrographs of the films.^28,38^

X-ray photoelectron spectroscopy (XPS) characterizations were performed in a Phoibos 100 DLD X-ray spectrometer from SPECS. Before analysis, samples underwent pretreatment via Ar ion bombardment (10^–6^ mbar, 5 kV, 5 min). The spectra were collected in the pass energy constant mode at 50 eV using an Mg Kα source. C 1s signal at 284.8 eV was used to calibrate the spectra’s binding energy (BE). The assignment of the BE to the different elements in the spectra corresponds to the data in the literature.^39^

Optical transmittance properties of the samples deposited on fused silica substrates have been analyzed in the 200–2500 nm wavelength range recorded in a PerkinElmer Lambda 750 S UV–vis–NIR spectrophotometer. In situ measurements were carried out with a homemade device to characterize hysteresis curves. It consisted of two ceramic heater-resistor holders with a concentric pierced 5 mm diameter hole at the center, allowing light transmission, and was used to hold the samples during the spectra acquisitions. The plates were connected to an ISOTECH IPS-405 dc (DC) power source, allowing uniform sample heating from both sides. Additionally, one of the heating plates was equipped with an encapsulated thermocouple placed close to the samples and connected to a temperature reader. The transmittance versus temperature thermochromic hysteresis loops were obtained at a wavelength of 2000 nm in variable steps to temperature, ranging from room temperature (RT) to 110 °C in air. The current was gradually applied until the target temperature was reached. After achieving each desired temperature, an equilibration period was observed to attain thermal equilibrium and compensate for any minor temperature fluctuations. Raman spectroscopy characterizations were carried out in a Horiba Jobin-Yvon LabRAM spectrometer equipped with a confocal microscope with a 50× objective and a green laser of 532 nm wavelength. The spectral resolution for this configuration was ∼1.7 cm^–1^. No polarization was applied during the experiments. Low laser powers were utilized to prevent local heating that could lead to oxidation of the VO_2_ films and unintended phase transitions.

## 3 Results and Discussion

### 3.1 Synthesis of V_2_O_5_ Films

Figure 1a resumes the procedure for synthesizing aerogel-like V_2_O_5_ thin films consisting of one or several cycles of combined VOPc RPAVD of vanadium-containing plasma polymers and remote soft plasma etching. The VOPc plasma polymer films present an intense light absorption related to the VOPc bands, as shown in Figure S1a, and present a continuous and homogeneous microstructure (Figure S1b), with stoichiometry similar to the precursor molecule. In each cycle, the VOPc plasma polymer acts as a sacrificial layer for the ultraporous V_2_O_5_ films by plasma etching. Thus, the V-containing plasma polymer conformally coats the substrate surface (C_1_ cycle) or the previously deposited porous oxide films (C_2_–C*_n_* cycles) before being fully oxidized (see the schematic in Figure 1a). The oxygen plasma etches the organic part of the polymer films, generating low-dense inorganic V_2_O_5_ films by the oxidation of the V^4+^ cations of the VOPc films. Simultaneously, carbon and nitrogen elements of the VOPc polymer form oxygenated volatile species that are pumped out of the chamber. This deposition procedure is analogous to the recently reported method for synthesizing aerogel-like partly crystalline TiO_2_ optical films using a Ti­(IV) phthalocyanine precursor.^28^ A key factor controlling the final porous microstructure is the substrate temperature during the etching step, as it governs both the extent of polymer oxidation and the stability of the growing framework. For this reason, a processing temperature of 120 °C was selected. In contrast, variations in plasma power, gas ratio, or pressure have only a minor effect as long as the sacrificial polymer is fully removed. Although room-temperature plasma etching is possible, it restricts the thickness of the intermediate sacrificial layers to only a few tens of nanometers.^28^

**Figure 1:**
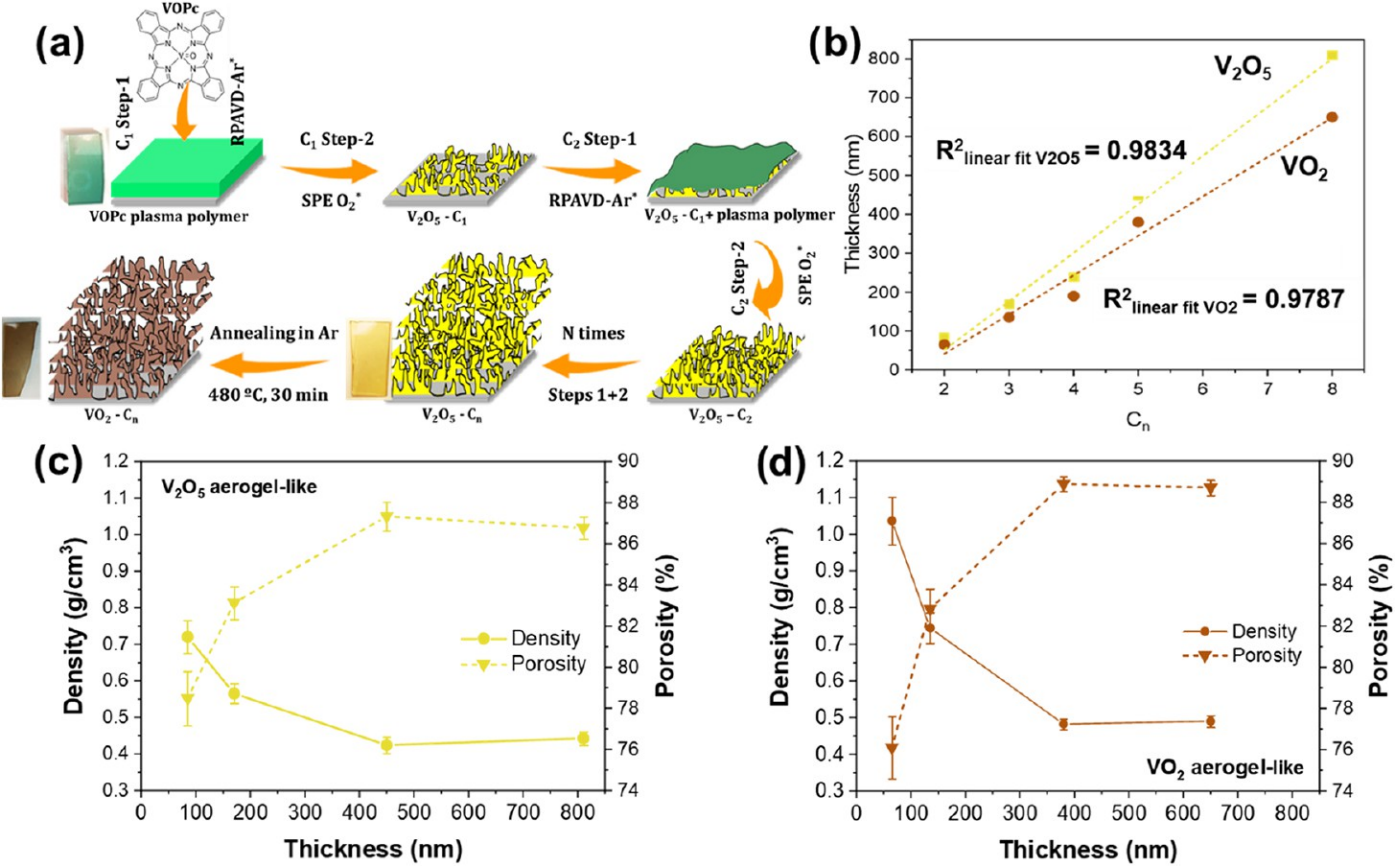
(a) Scheme of the cyclic procedure for fabricating aerogel V_2_O_5_ and VO_2_ films. (b) Relation between film thickness and the number of cycles for aerogel-like V_2_O_5_ films and the corresponding VO_2_ aerogel-like after annealing. The V_2_O_5_ films were synthesized in each cycle using sacrificial V-containing plasma polymers of ∼200 nm. (c, d) Density and porosity values as a function of film thickness for V_2_O_5_ (c) and VO_2_ aerogel-like films (d), these latter after the thermal annealing of films in (c).

The film thickness exhibits quasi-linear growth with the number of deposition and etching cycles, as shown in Figure 1b. The thickness reduction observed after Ar thermal annealing, which transforms the V_2_O_5_ to VO_2_, is consistent with the expected change in bulk densities between these two oxides (ρ_V_2_O_5__ = 3.36 g·cm^–3^; ρ_VO_2__ = 4.34 g·cm^–3^).^40^ Figure 1c shows the evolution of V_2_O_5_ film density deposited by RPAVD-Ar/SPE procedure as a function of the layer thickness. The figure also shows the corresponding porosity values calculated using the relationship Γ(%) = 100·(1 – ρ/ρ_r_), taking the density of reference crystalline V_2_O_5_ as ρ_r_ = 3.36 g·cm^–3^.^40^ The first RPAVD-Ar/SPE cycle yielded films with a density as low as 0.72 g·cm^–3^ and a porosity of 78.5%. As the layer thickness increases, the density decreases dramatically, reaching a value of 0.42 g·cm^–3^ at 450 nm of thickness, which corresponds to a film porosity as high as 87.3%. Further increases in thickness led to slight changes in these values, with the density increasing to 0.44 g·cm^–3^ and the porosity increasing to 86.8% at a thickness of 810 nm. Thus, the density and porosity values obtained are in the range of those reported for V_2_O_5_ aerogels obtained by supercritical drying methods^41^ and V_2_O_5_ aerogel thin films deposited by sol–gel.^32,53^ For this reason, we refer to them hereafter as aerogel-like V_2_O_5_ films.

Figure 2a showcases the planar and cross-sectional films of V_2_O_5_ aerogel-like thin films deposited on Si (100) that correspond to the synthetic cycles C1–C3 and C5 using VOPc sacrificial layers of ∼200 nm per cycle. From the first cycle, the films present a unique, low-dense sponge-like microstructure of interconnected percolated oxide structures surrounding a homogeneous distribution of quasi-circular pores with a high void fraction. Note that all the solid oxide structures in the micrographs are highly porous and fully interconnected. Figure 2c also shows that such a unique porous structure is interconnected. As the number of cycles increases, the size of the porous increases, retaining the sponge-like structure and forming an expansive network where empty spaces prevail. This open porous low-density structure is congruent with the measured density values shown in Figure 1c. Notably, compared with aerogel-like TiO_2_ films prepared by a similar procedure,^28^ the fully interconnected foam-like structure of the vanadium oxide films is distinctive.

**Figure 2:**
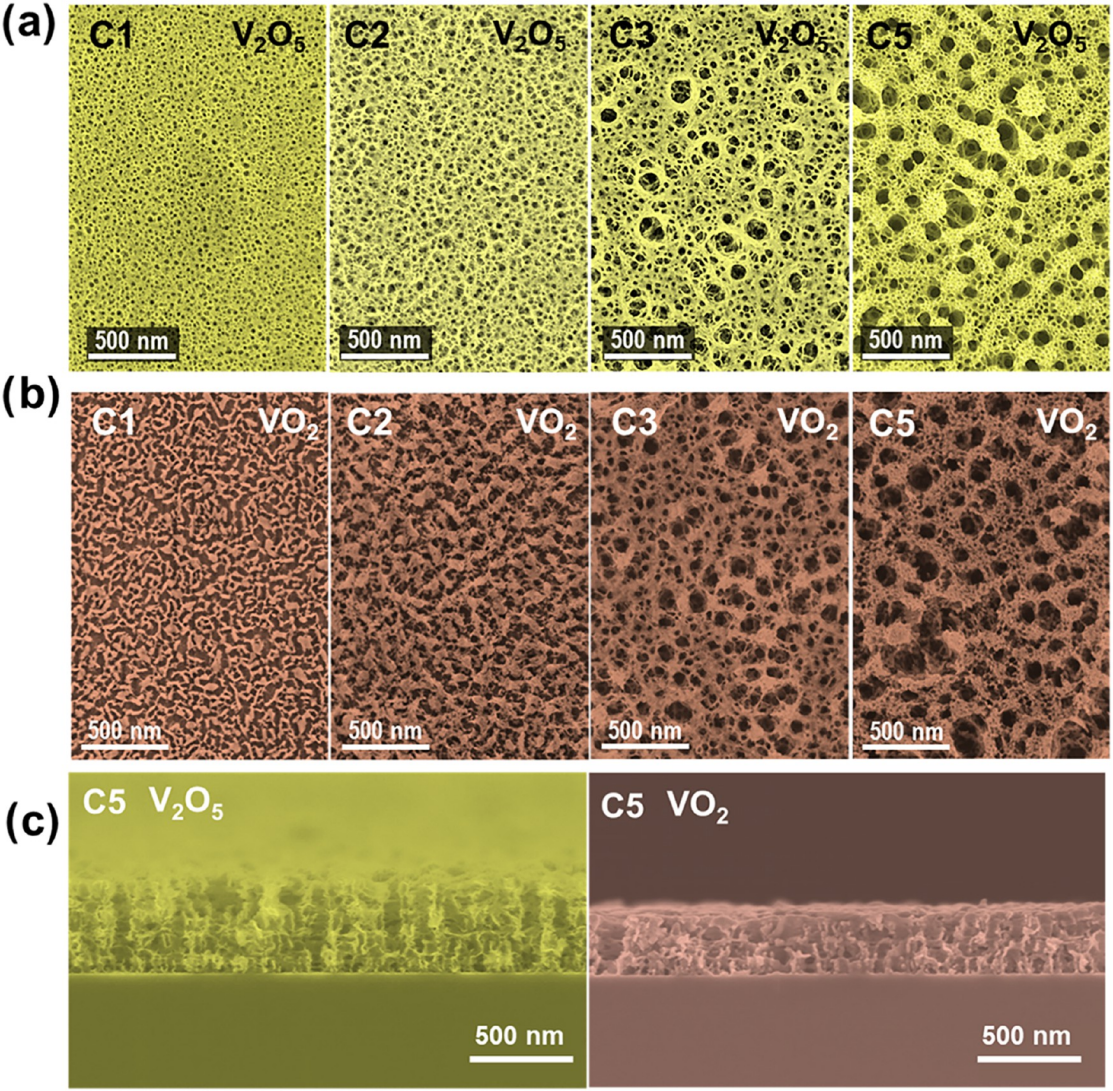
(a) Normal view FESEM micrographs of V_2_O_5_ aerogel-like films corresponding to cycles C1 to C5. (b) Normal view FESEM micrographs of VO_2_ obtained after annealing. (c) Cross-sectional SEM micrographs of a C5 aerogel-like thin film, showing before (left) and after (right) annealing.

Figure 3a shows FESEM micrographs corresponding to V_2_O_5_ films synthesized by RPAVD-O_2_ with 40 and 110 nm thicknesses, respectively. The samples display a homogeneous and featureless microstructure in the thickness range studied. Samples fabricated by this technique are limited to lower thicknesses than aerogel-type samples due to their characteristic optical properties (i.e., high absorptance in the visible and NIR range, as will be shown in the following sections). These films will be used as a reference for properly comparing the optical properties of the high porosity layers synthesized by the cyclic plasma polymerization and plasma etching procedure.

**Figure 3:**
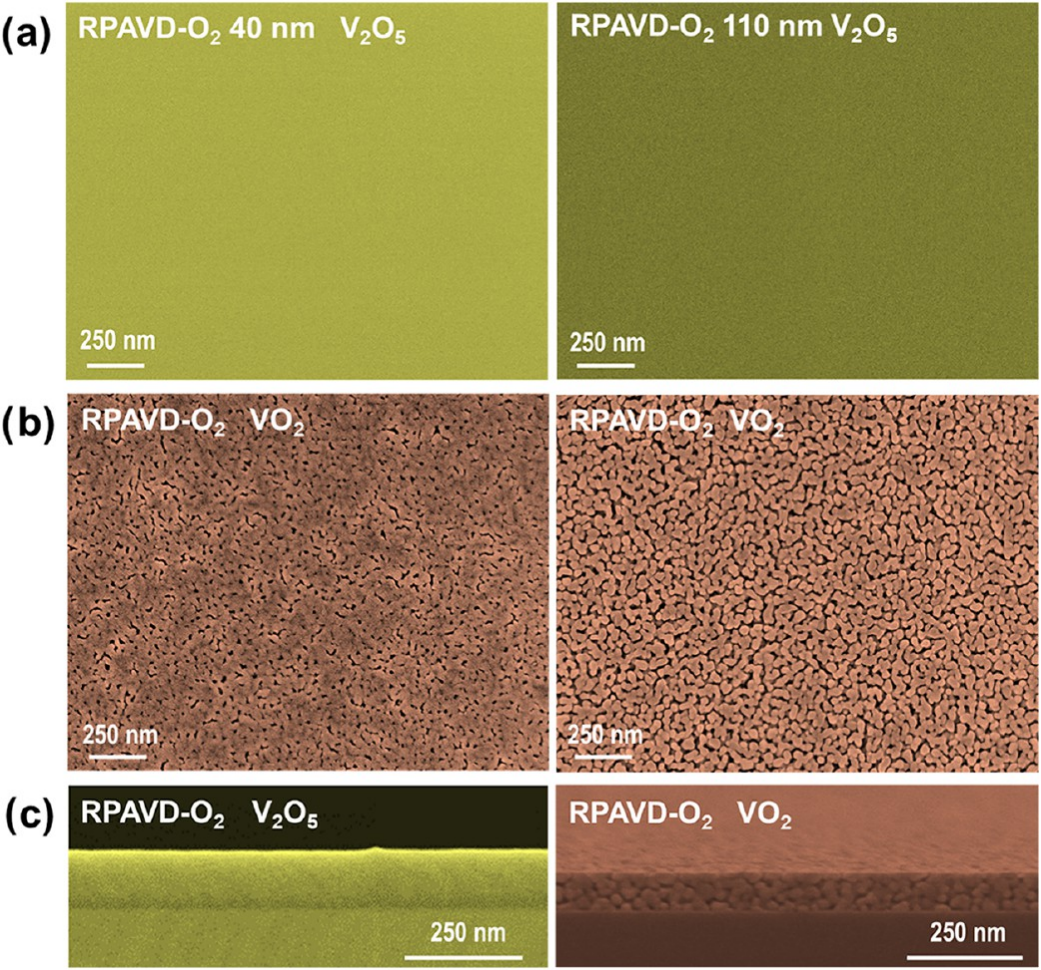
(a) FESEM images of two V_2_O_5_ films of 40 nm (left) and 110 nm (right) films deposited by RPAVD-O_2_. (b) FESEM images of the thin films in (a) after thermal annealing. (c) Cross-sectional FESEM micrographs of the RPAVD-O_2_ 110 nm V_2_O_5_ and annealed VO_2_ films in (a, b), respectively.

### 3.2 Synthesis of VO_2_ Films by Annealing

The following experimental step after the film synthesis consisted of determining the conditions for the thermal reduction of the plasma-deposited V_2_O_5_ films to get thermochromic VO_2_ films. The criterion selected to determine the optimum annealing treatment conditions was to maximize the thermochromic response of the samples, that is, to maximize the difference between the light transmission in the infrared region below and above the transition temperature. Note that a complete analysis of the thermochromic response of the films will be presented in the next section. Figure 4a shows the normalized transmittance variation between room temperature and 90 °C (to be sure that the temperature is well below and above the *T*_t_), at λ = 2000 nm, of a plasma-deposited V_2_O_5_ film subjected to annealing at different temperatures for 0.5 h (under Ar atmosphere). It is observed that below 400 °C, the transformation from V_2_O_5_ to VO_2_(M) is negligible. Above this temperature, the formation of VO_2_ (M) crystals becomes evident. The transmittance variation (note that this Δ*T* is normalized) increases as the annealing temperature rises above 400 °C, reaching a peak at 480 °C. At temperatures exceeding 480 °C, the transmittance variation decreases, very likely due to the transformation of VO_2_(M) into more reduced species.

**Figure 4:**
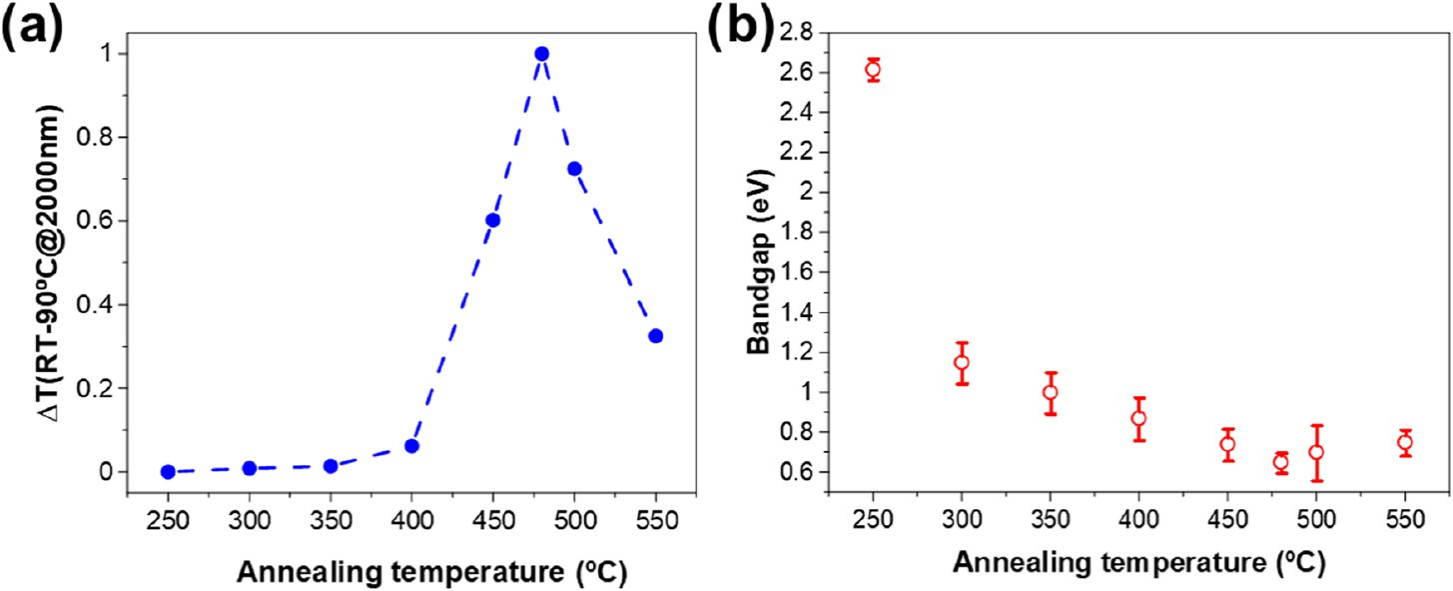
(a) Normalized transmittance variation at λ = 2000 nm between room temperature and 90 °C for as-deposited V_2_O_5_ aerogel-like samples annealed at different temperatures. The annealing time at each temperature was 0.5 h. (b) Calculated optical bandgap energy by the Tauc-plot method of the V_2_O_5_ aerogel films in (a) as a function of the annealing temperature.

Figure 4b illustrates the evolution of the bandgap for a V_2_O_5_ aerogel-like film with temperature calculated using the Tauc-Plot method^42^ as a function of the annealing temperature. As the temperature rises, the bandgap value decreases gradually, ascribed to the transformation from V_2_O_5_ to VO_2_ (the latter having a bandgap between 0.6 and 1.0 eV).^43,44^ The band gap reaches 0.6 eV at 480 °C, confirming this as the optimal annealing temperature. Its gradual evolution indicates that microstructural and chemical changes start at lower temperatures before affecting the thermochromic transition.

As mentioned before, when the V_2_O_5_ aerogel samples undergo the annealing process, there is an approximate 20% reduction in the thickness of the films, regardless of the initial value (see Figure 1b). Figure 2b shows the FESEM images of the films after the thermal annealing. Compared with the corresponding V_2_O_5_ aerogel-like films, a modification in the microstructure of the newly formed VO_2_ films is observed after the annealing, leading to more aggregated oxide structures. In the thinner films (C_1_ and C_2_), the aggregation of the solid part results in a higher volume of macroporous structures. However, in the case of the thicker films (C_3_ and C_5_), the aggregation of the oxide structures is also observed, but the films present a pore distribution and oxide structures percolation that resembles that of the starting V_2_O_5_ films. Figure 1d shows the evolution of film density and porosity after the annealing process. The porosity values are determined considering a reference density for VO_2_ of 4.34 g·cm^–3^.^40^

The density values are, in all cases, higher than those of the corresponding V_2_O_5_ films (Figure 1c). Thus, from an initial value of 0.72 g·cm^–3^ for the thinner film, 1 g·cm^–3^ is reached for the annealed film. The density of the VO_2_ films decreases as the thickness of the films increases while remaining, in all cases, above the values of the starting V_2_O_5_ oxide films. The values for the thicker layers are 0.48 and 0.49 g·cm^–3^ for the 380 and 650 nm films, respectively. The porosity values are relatively high, reaching 82.8% for the 135 nm thick sample and higher than 88.5% for the thicker layer. These values are in the range of aerogel materials. Note that the higher porosity values of the VO_2_ films after annealing depend on the density value of the VO_2_ reference used in the calculation, which is higher than that of the V_2_O_5_ reference.

Contrarily, when the reference RPAVD-O_2_ V_2_O_5_ samples are annealed to get VO_2_ thin films (Figure 3b), the microstructure of the films undergoes a significant transformation. The resulting structure manifests as a rugged surface morphology comprising minute irregular granules ranging in size from 35 to 70 nm. This aggregated structure is more pronounced as the thickness increases. The cross-sectional view in Figure 3c shows a similar aggregated microstructure in the film cross-section. Besides, an open porous structure between the solid aggregates is also patent in the films, being more pronounced for the thickest film.

### 3.3 Thin Film Crystallinity and Surface Composition

Figure 5a shows transmission electron microscopy (TEM), HAADF-STEM, SAED pattern, and HRTEM micrographs of as-deposited V_2_O_5_ aerogel-like film. These images reveal an ultraporous structure consisting of roughly spherical cavities with wall thicknesses ranging from 5 to 10 nm and crystalline domains in the range 4–8 nm. The sample exhibits both amorphous and nanocrystalline phases, as indicated by the broad and diffuse halo and the diffraction rings observed in the SAED pattern and by the lattices fingers and amorphous regions visible in the HRTEM image. After annealing at 480 °C (Figure 5b), the sample still exhibits a high degree of porosity, as observed in the representative TEM micrograph, which also reveals elongated crystals with the shortest dimensions in the range ∼10–20 nm. The SAED pattern (inset of Figure 5b) confirms the formation of a polycrystalline structure.

**Figure 5:**
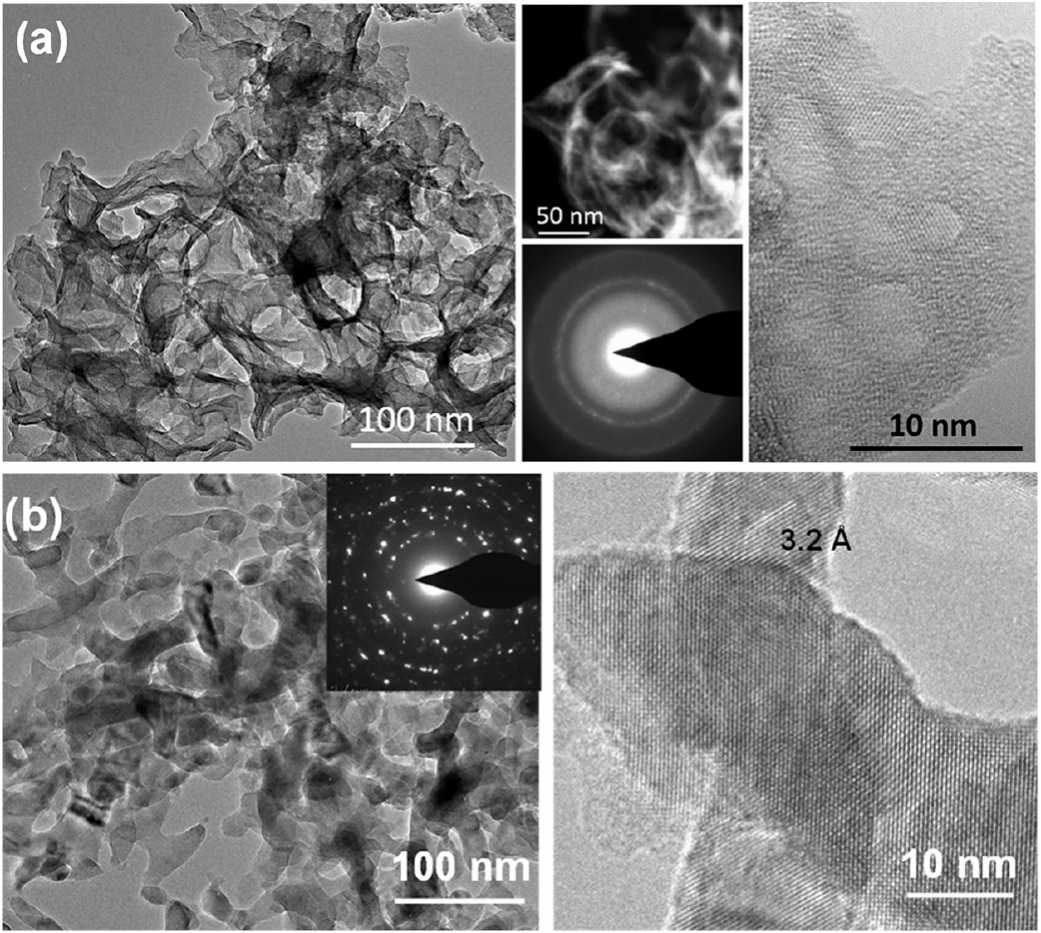
(a) TEM micrograph (left), HAADF-STEM image and SAED pattern (center), and HRTEM micrograph (right) of an aerogel-like V_2_O_5_ film. (b) TEM image, SAED pattern (left), and HRTEM micrograph (right)­of a VO_2_ film after annealing.

The energy loss near-edge structure (ELNES) of the oxygen K-edge in the EELS spectra in vanadium oxide provides a valuable insight into the local density of states (LDOS) at the oxygen site, serving as a reliable indicator of the material’s oxidation states.^36,45^ Changes in the vanadium valence state lead to a relative chemical shift between the oxygen K-edge and vanadium L3-edge. The ELNES features and the relative chemical shifts for standard V_2_O_5_(V^5+^) and VO_2_(V^4+^), have been well documented in the bibliography.^36^ Figure 6 compares the EELS spectra from an aerogel-like VO_2_ sample with those of V^5+^ and V^4+^ references (see Section 2). The aerogel-like film in the figure has a distance between V-L_3_ and O–K e_g_ peaks (Δ*E*) and relative intensities of the O–K t_2g_ and e_g_ peaks that are similar to the VO_2_ reference values,^36,45^ which is consistent with the formation of VO_2_ after the annealing. Note that the V_2_O_5_ reference presents 1 eV less in the value of Δ*E* (13 vs 14 eV), as reported in literature.^36,45^

**Figure 6:**
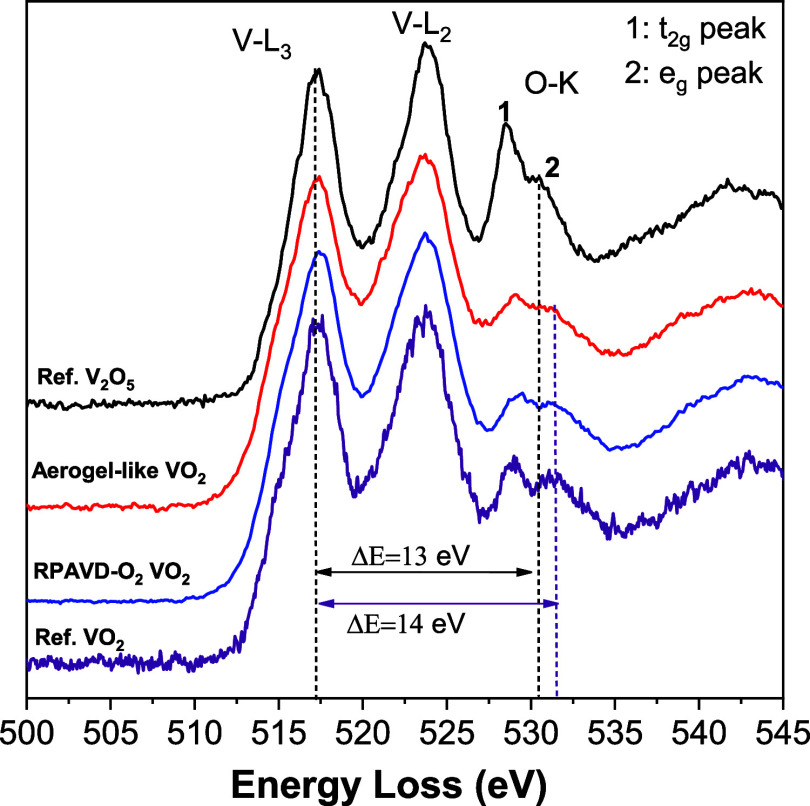
Energy loss near edge structure (ELNES) of aerogel-like VO_2_ and RPAVD-O_2_ samples and VO_2_ and V_2_O_5_ reference spectra. The energy separation (Δ*E*) between the O K-edge (e_g._, peak) and the V-L_3_ edge for the two reference samples is indicated in the figure.

Figure 7a shows SAED patterns of the aerogel-like VO_2_ film. The left pattern corresponds to the as-prepared state, while the central and right patterns were obtained after progressive heating under the electron beam. The ring spacings measured in the initial pattern (left) correspond to the monoclinic VO_2_(M) phase, consistent with the XRD analysis (ICSD No. 60767). However, the measured values are slightly larger, suggesting an increased lattice parameter and indicating lattice strain. Specifically, the distance for the first diffraction ring is measured at 3.5 Å, compared to the theoretical 3.2 Å for the (01̅1) plane (ICSD No. 74705). Upon beam-induce heating, this spacing decreases to 3.4 Å (central pattern) and finally to 3.2 Å after prolonged exposure (right), indicating a transition. The decreased distances in the right pattern of Figure 7a, can be attributed to the rutile VO_2_(R) phase (ICSD No. 66665). Figure 7b displays the evolution of the diffraction patterns with increasing electron beam irradiation time and heating of the sample.

**Figure 7:**
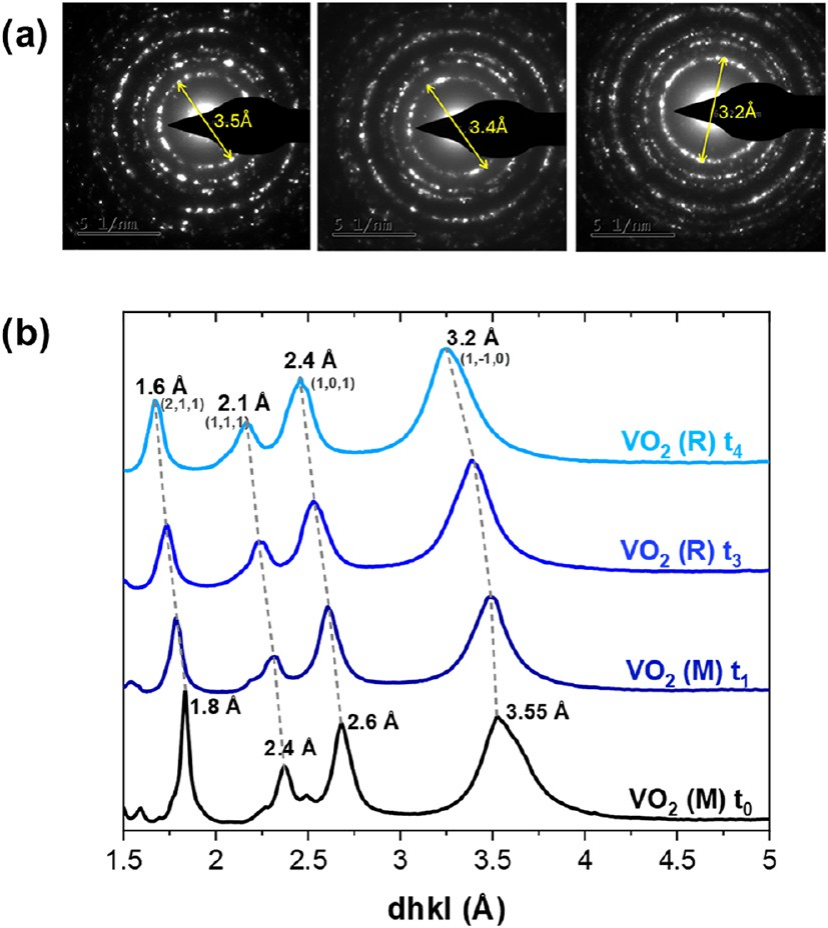
(a) SAED of the aerogel-like VO_2_ film heating by electrons by increasing time. The diffraction patterns correspond to the initial state (left) and after heating under the electron beam (center) and (right). (b) Plot of the intensity of the diffraction rings in (a) for increasing periods from *t*_0_ to *t*_4_.

TEM analysis of an RPAVD-O_2_ V_2_O_5_ sample (Figure 8a) reveals that the film comprises packed oxide nanocrystals ranging from 20 to 60 nm in size. The SAED pattern shows rings characteristic of polycrystalline samples with no evidence of an amorphous phase. The diffraction rings can be assigned to V_2_O_5_. After annealing (Figure 8b), the sample is still composed of nanocrystals with a slight increase in crystal size to approximately 30–90 nm. The rings′ spacings in the figure correspond to VO_2_, indicating a phase transformation. Furthermore, the ELNES spectrum of this film (Figure 6) confirms that, after annealing, the film predominantly contains V^4+^ species.

**Figure 8:**
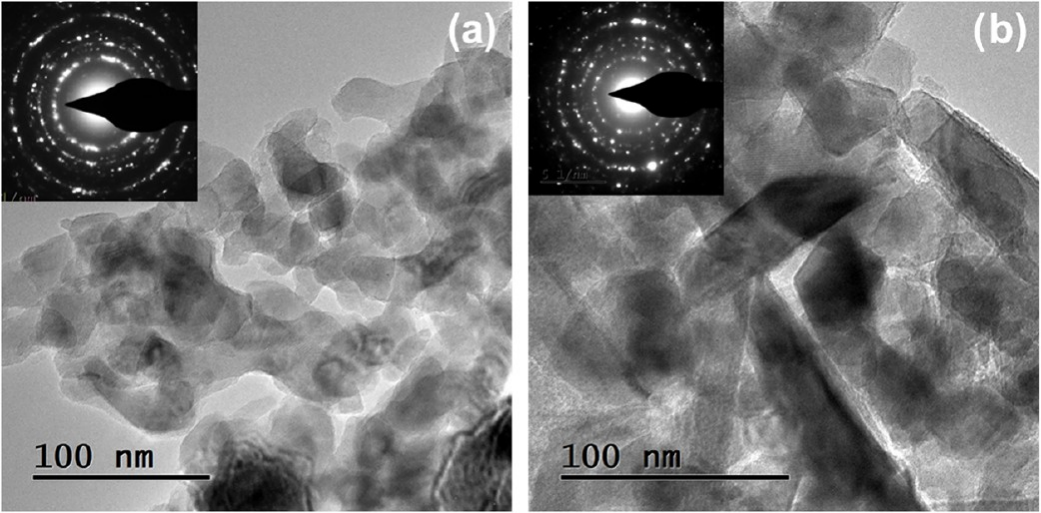
TEM micrographs and SAED of an RPAVD-O_2_ film (a) before and (b) after annealing to induce the transformation to VO_2_.

Grazing incidence X-ray diffraction (GIXRD) analysis was performed to evaluate the crystallinity of the samples. Figure 9a displays the GIXRD diffractograms for a 450 nm aerogel-like V_2_O_5_ film and the corresponding VO_2_ film after annealing. The diffractograms show that both types of films are crystalline, showing the transition from orthorhombic V_2_O_5_ to monoclinic VO_2_ after the annealing, similar to what was observed by HRTEM. In both cases, the width of the XRD peaks can be attributed to the finite crystallite size, which influences the peak broadening according to the Scherrer equation. This broadening reflects the minimum crystallite size within the sample, as smaller crystallites produce wider peaks.^46^ The as-deposited V_2_O_5_ aerogel-like sample exhibits well-defined peaks at lower angles between 15° and 35°. These peaks correspond to the planes (020), (001), (110), (031), and (130), characteristic of the orthorhombic structure of α-V_2_O_5_ (ICSD No. 60767, *Pmmn*, *a* = 11.5 Å, *b* = 3.5 Å, *c* = 4.3 Å and α = β = γ = 90°). According to the Scherrer equation, the crystallite size for the V_2_O_5_ aerogel is estimated to be 6.5 nm. This crystalline size is consistent with the HRTEM results shown in Figure 5a, where small crystals of around 5 nm (note that the walls were as thin as 5–10 nm) were surrounded by an amorphous matrix. These results further demonstrate that the cycling plasma polymerization and etching can give rise to crystalline V_2_O_5_ thin films at low temperatures (i.e., 120 °C during the etching process in each cycle) without requiring high temperatures to complete the film oxidation.

**Figure 9:**
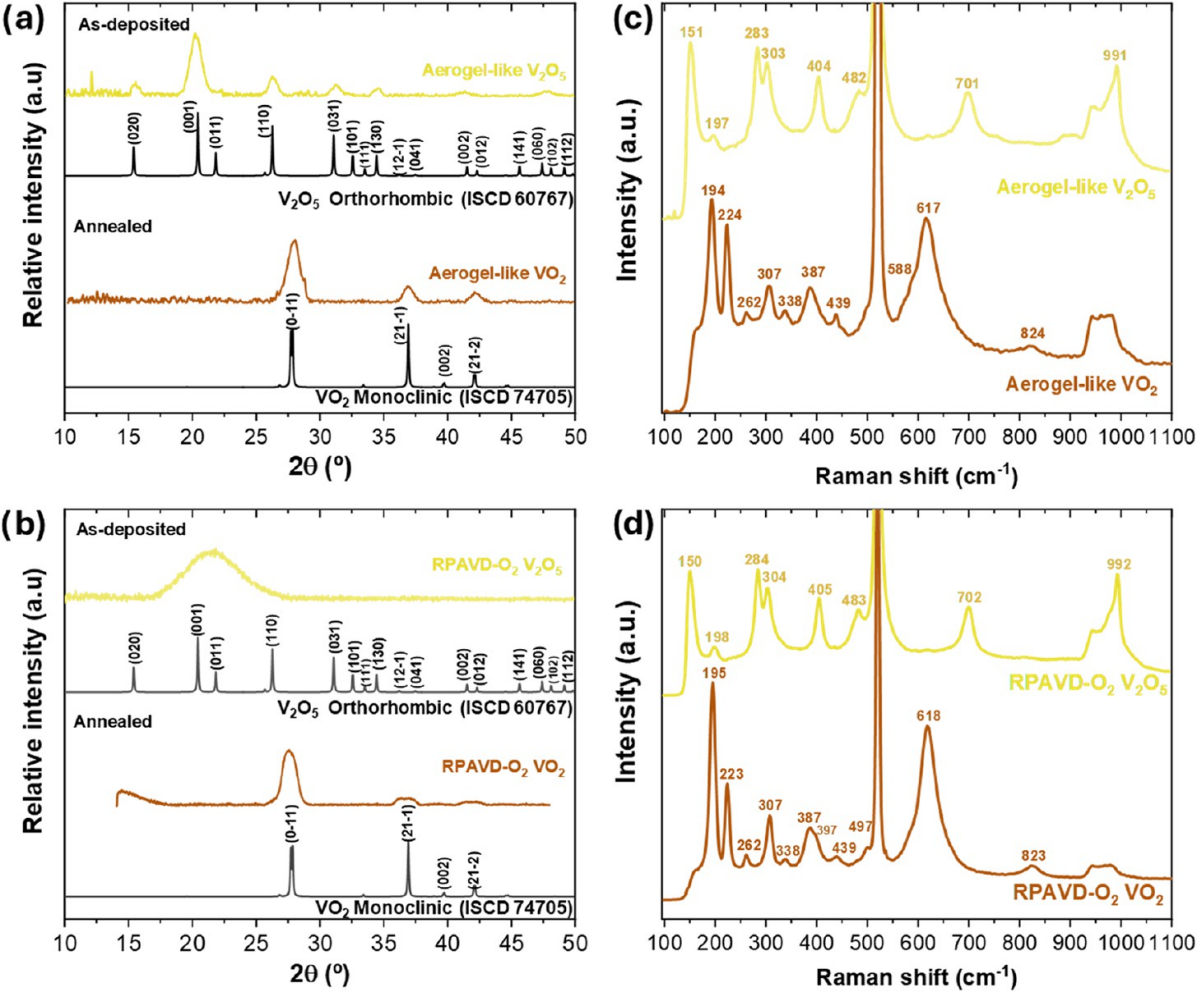
(a) GIXRD patterns of an as-deposited 450 nm thick V_2_O_5_ aerogel-like film and the same film after thermal annealing. (b) GIXRD of a 110 nm thick V_2_O_5_ film deposited by RPAVD-O_2_ before and after annealing. The GIXRD references of V_2_O_5_ and VO_2_ in (a, b) are included for comparison. (c) Raman spectra of a 450 nm thick aerogel-like V_2_O_5_ film before and after annealing. (d) Raman spectra of a 110 nm thick RPAVD-O_2_ film before and after annealing. The peak at ∼520 cm^–1^ from the Si(100) substrate is visible in the Raman spectra.

After annealing, the samples completely lose the diffraction peaks of the V_2_O_5_ and develop mainly four additional peaks that correspond to the (01̅1), (211̅), (002), and (212̅) planes, indicative of the monoclinic structure of VO_2_ (M1) (ICSD No. 74705, *P*2_1_/*c*, *a* = 5.7 Å, *b* = 4.5 Å, *c* = 5.4 Å nm, α = γ = 90° and β = 122.61°). According to the Scherrer equation, the crystallite size for the VO_2_ aerogel after annealing is estimated to be 6.6 nm, which is also in good agreement with the crystalline sizes observed by HRTEM (10–20 nm). Importantly, no evidence can be observed for other crystalline phases, such as V_2_O_5_, V_6_O_13_, or V_2_O_3_, indicating the high purity of the films. These results constitute compelling evidence validating the optimization of the annealing process for VO_2_ film production.

Figure 9b shows the GIXRD of a VO_2_ sample prepared by RPAVD-O_2_ of 110 nm after and before the annealing process. The reference V_2_O_5_ porous samples (Figure 9b) do not show any diffraction peaks. After annealing, the resulting VO_2_ film is crystalline, showing peaks similar to those of the aerogel-like VO_2_ films corresponding to the monoclinic phase without the presence of additional peaks that could be ascribed to other vanadium oxide phases. The lack of diffraction patterns of the V_2_O_5_ films may be attributed to the low thickness of the analyzed samples because the HRTEM analysis presented in Figure 8 showed that the sample is nanocrystalline.

The vanadium oxide films were characterized by room temperature Raman spectroscopy before and after annealing and analyzed according to the literature.^47–50^ Further details about the Raman characterization are provided in Supporting Information S2. As depicted in Figure 9c, the as-deposited aerogel-like thin film exhibits characteristic peaks indicative of pure crystalline α-V_2_O_5_, congruent with the previous XRD and TEM characterizations, with no observable traces of other vanadium oxides or compounds.

The Raman spectrum for the aerogel-like annealed sample displays all the peaks attributed to VO_2_(M1), consistent with previously reported data.^47,48^ Importantly, no other phases or polymorphs were detected. The slight discrepancies between our data and those in the literature refer to small shifts in the Raman peak positions (in cm^–1^). These shifts can be attributed to the pronounced difference in thermal expansion coefficients between VO_2_ and the silicon substrate, which induces heightened internal strain in films deposited on this substrate, thus explaining the observed variations.^49^ The low-frequency phonons at 194 and 223 cm^–1^ are associated with lattice motion involving V–V bonds, while the remaining peaks correspond to vibrational modes of V–O bonds. The bands in the low wavenumber region (<400 cm^–1^) are attributed to V–O–V bending modes. As the wavenumber transitions to the intermediate range (400–800 cm^–1^), the bands correspond to V–O–V stretching modes. The broad peak at 613 cm^–1^ is a convolution of the 588, 613, and 661 cm^–1^ peaks. The bands in the high wavenumber range (>800 cm^–1^) can be assigned to V=O stretching modes indicative of distorted octahedra and square pyramids.

The interpretation of the Raman spectra of both RPAVD-O_2_ as-deposited and annealed is similar, presenting Raman bands corresponding to crystalline V_2_O_5_ and VO_2_, respectively (Figure 9d). Thus, it is noticeable that there is a discernible level of crystallinity in the as-deposited porous sample that the GIXRD analysis (Figure 9b) could not capture, likely due to the low thickness and random orientation of the nanocrystallites with few diffraction planes.

The surface composition of the as-deposited and annealed vanadium oxide aerogel-like films was studied by XPS and analyzed according to the literature.^39,50^ Figure 10a shows the core-level V 2p of an aerogel film before and after annealing. The as-deposited aerogel-like film presents exclusively V^5+^ bands at ∼517.6 eV (V^5+^, 2p_3/2_) and ∼524.9 eV (V^5+^, 2p_1/2_) without traces of other vanadium species. This result indicates that the remote oxygen plasma treatment effectively oxidized the vanadium species (mainly V^4+^) from the VOPc sacrificial plasma polymer to their highest oxidation state. The VO_2_ aerogel-like sample obtained after the annealing treatment shows two majority V^4+^ core level bands at ∼516.4 eV (V^4+^, 2p_3/2_) and ∼523.7 eV (V^4+^, 2p_1/2_) in the same binding energies and two additional less intense bands at the V^5+^ binding energies. Considering the results of the previous GIXRD, HRTEM, and Raman characterizations, the small percentage of V^5+^ at the surface of the annealed films is likely due to surface oxidation. The RPAVD-O_2_ films present surface compositions similar to those of the aerogel-like films after and before the annealing.

**Figure 10:**
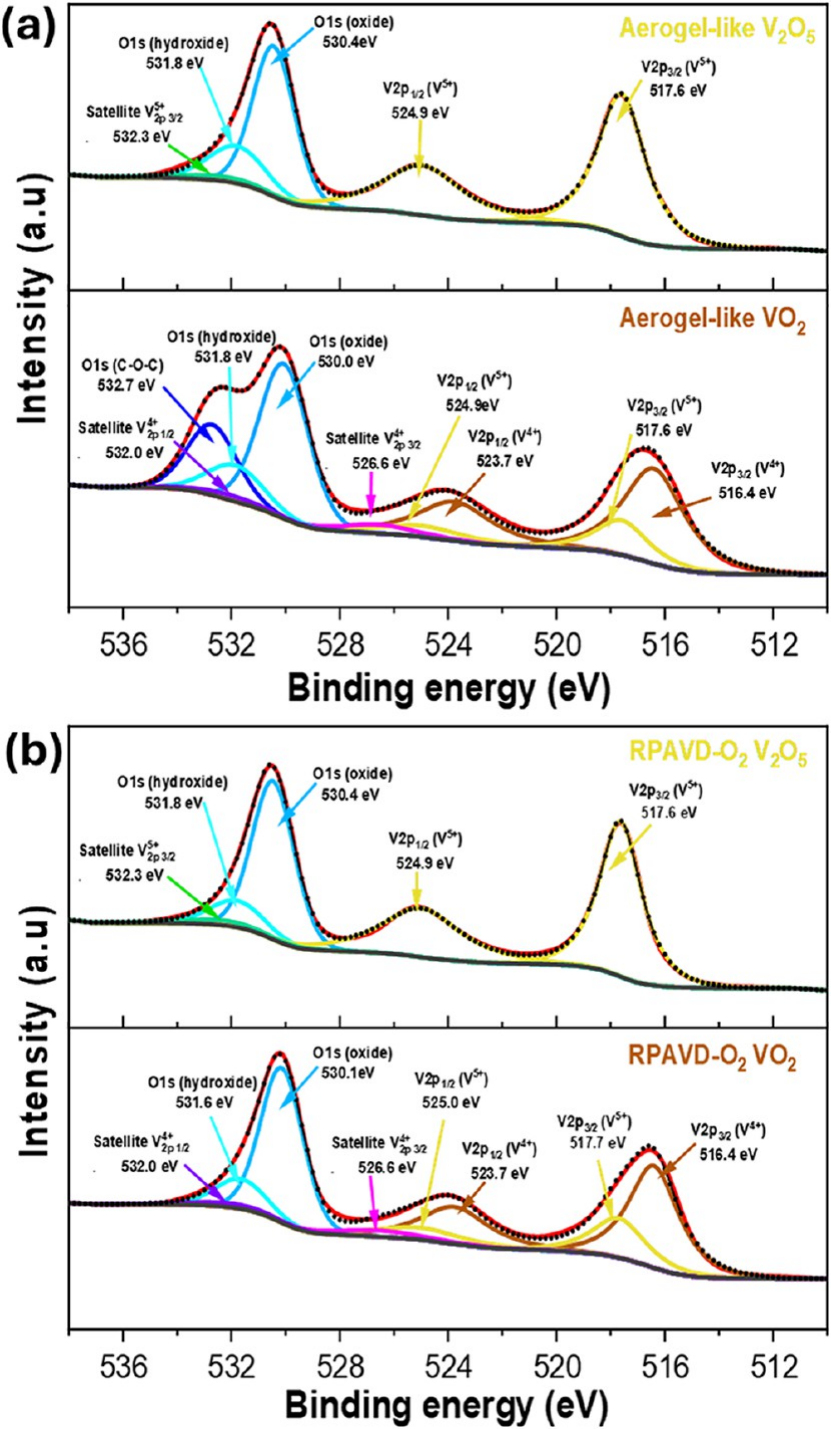
V 2p + O 1s XPS spectra for (a) aerogel-like and (b) RPAVD-O_2_ films before and after the annealing process.

Additional details about the XPS analyses of the RPAVD-Ar VOPc sacrificial plasma polymers and sublimated samples, and the fitting parameters used for the characterization of the oxide films, are included in Supporting Information S3 (Figure S2 and Tables S1 and S2).

### 3.4 Optical Performance

The thermochromic performance of the samples was studied by UV–vis–NIR transmittance spectroscopy at controlled temperature for samples deposited on fused silica.

Figure 11a top shows the results of three aerogel-like samples measured at room temperature (25 °C) and 90 °C. The overall shape of the spectra is characteristic of VO_2_.^51,52^ The films exhibit strong UV absorption and visible transparency at room temperature, reaching transmittance values of 85–63% at 630 nm, depending on the layer thickness. Besides, the aerogel-like sample presents a high transparency in the NIR region, with transmittance values at λ = 2000 nm in the 93–89% range, depending on the layer thickness. All the samples have transmittance values higher than 90% at 2500 nm. At 90 °C, the aerogel-like VO_2_ samples show a significant reduction of the IR transmittance due to the transition from the insulating to the metallic phase, reaching values at λ = 2000 nm in the 74.4–38.4% range, depending on the thickness.

**Figure 11:**
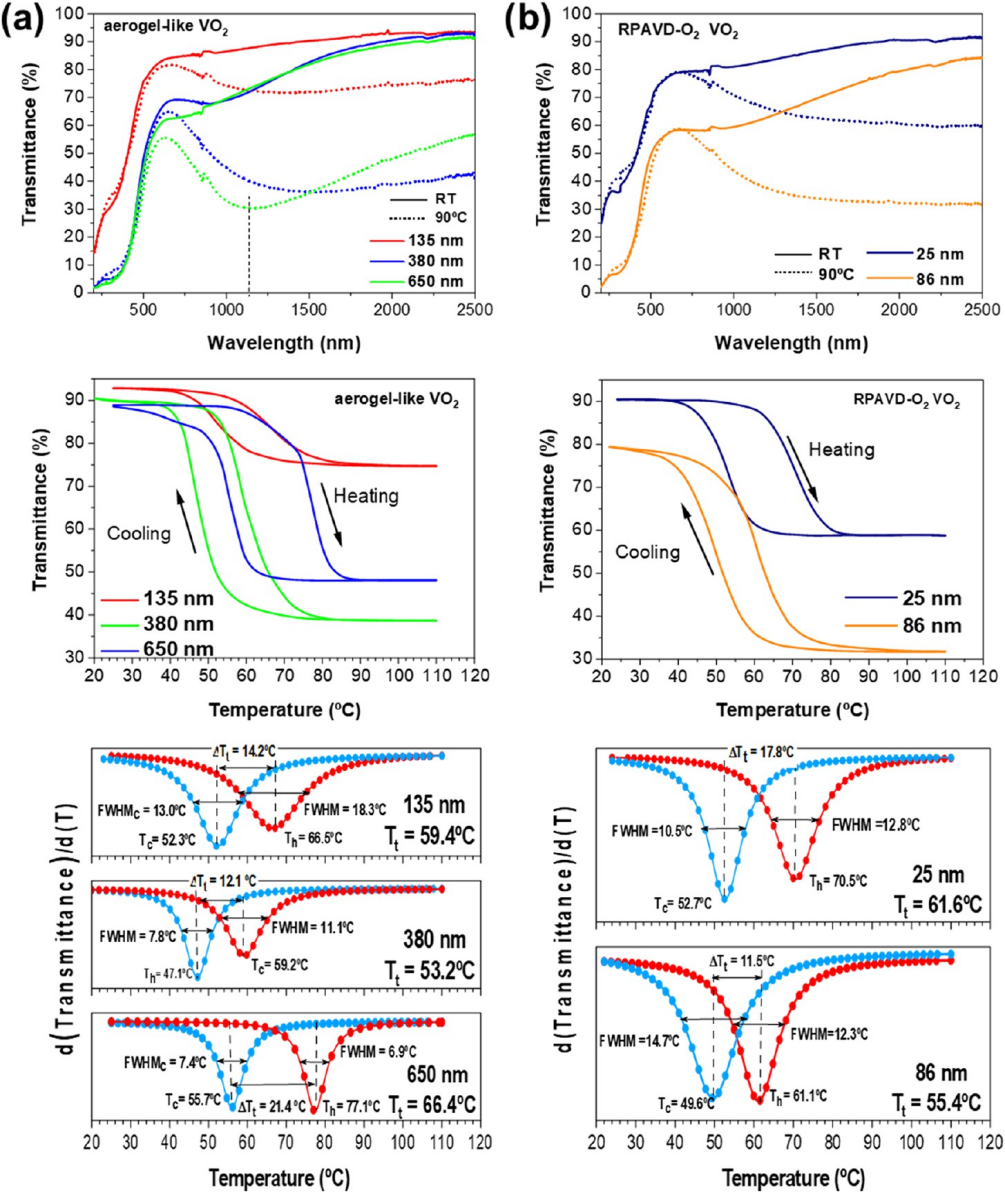
Temperature-dependent transmittance spectra in the UV–vis–NIR region at 25 and 90 °C (top), measured hysteresis loops (middle), and first derivative (d­(*T*_f_)/d­(*T*)) versus temperature (bottom) for a set of aerogel-like VO_2_ thin films (a) and RPAVD-O_2_ VO_2_ thin films (b).

For the assessment of the films’ potential for smart window application, we have calculated their key thermochromic parameters. These include luminous transmittance (*T*_lum_), solar modulation (Δ*T*_sol_), and near-infrared modulation (Δ*T*_IR_), all derived from the transmittance values at each thickness. These properties are quantified according to the following expression [1]­

1 $$T_{i}=\frac{\int T\left(\lambda \right)\phi_{i}\left(\lambda \right)d\lambda }{\int \phi_{i}d\lambda }$$

and Δ*T*_*i*_ **=* T*_*i*,cold_ – *T*_*i*,hot_. *T*(λ) represents the transmittance value for each wavelength; *i* denotes *lum*, *sol*, or *IR*; φ_lum_(λ) is the standard photopic efficiency of vision (spectral sensitivity of the human eye) in the range 380–780 nm, from values tabulated by the International Commission on Illumination.^53^ φ_sol_(λ) and φ_IR_(λ) are the solar irradiance spectrum for an air mass of 1.5 when the sun is at 37° above the horizon in the 280–2500 nm range and 780–2500 nm, respectively, with values tabulated by the American Society for Testing and Materials.^47^ Cold and hot values correspond to the curves measured at 25 and 90 °C after and before the thermochromic transition. All these results are gathered in Table 1.

**Table 1:** Optical Thermochromic Parameters during the Phase Transition of Selected Aerogel-like and RPAVD-O_2_ Samples^a^

			*T*_lum_ (%)			*T*_sol_ (%)			*T*_IR_ (%)		
sample	thickness (nm)	*T*_t_ (°C)	low *T*	high *T*	Δ*T*_lum_	low *T*	high *T*	Δ*T*_sol_	low *T*	high *T*	Δ*T*_IR_
aerogel-like	135	59.4	79.6	77.6	2.0	80.2	73.3	6.9	87.9	74.9	13.0
	190	58.5	77.3	74.7	2.6	78.0	70.8	7.2	85.8	72.6	13.3
	380	53.2	58.6	55.8	2.8	61.2	46.5	14.7	74.3	44.5	29.8
	650	66.4	54.4	49.2	5.2	58.6	39.8	18.8	73.4	37.9	35.5
RPAVD-O_2_	25	61.6	74.0	74.0	0.0	75.4	69.1	6.2	83.1	69.0	14.2
	85	55.4	54.0	52.4	2.4	54.8	44.5	10.3	63.8	42.2	21.6

a Low and high *T* correspond to 25 and 90 °C, respectively.

One intriguing aspect of the aerogel-like thermochromic films is their departure from the typical tens of nanometer thickness range of VO_2_ thermochromic compact films due to the inherent opacity of this material.^51,54^ As seen in Figure 11a, the thickness range of aerogel-like films is up to several hundreds of nanometers. The first notable behavior concerning *T*_lum_ is observed across all the aerogel-like samples. Generally, in low-thickness thermochromic films, the *T*_lum_ value at low temperatures is consistently higher than at high temperatures. Various studies have demonstrated that VO_2_ thermochromic films have a threshold thickness value beyond which the *T*_lum_ at high temperatures surpasses the T_lum_ at low temperatures. This reversal in Δ*T*_lum_ is attributed to interference effects^51,55,56^ and adversely affects Δ*T*_sol_, decreasing its value when the thickness exceeds the mentioned threshold. However, this phenomenon is not manifested in the aerogel-like samples, suggesting that the threshold thickness for the reversal in Δ*T*_lum_ has not yet been reached (see Figure 11a,top and Table 1). Thus, for aerogel-type samples, the Δ*T*_sol_ values for the thinnest films are 6.9% for a thickness of 135 nm, increasing by only 0.3% (i.e., to 7.2%) as the film thickness increases to 190 nm. Meanwhile, *T*_lum_ remained almost unchanged (79.6/77.6% and 77.3/74.7%, respectively, for low/high *T*). However, when the thickness doubles to 380 nm, the Δ*T*_sol_ value more than doubles, from 7.2% to 14.7%, with a *T*_lum_ value of 58.6/55.8% (low/high *T*). Further increasing the film thickness to 650 nm, the Δ*T*_sol_ value reaches a remarkable 18.7%, accompanied by a mild decrease in *T*_lum_ to just 54.4/49.2%. These findings demonstrate that aerogel-type samples exhibit an optimum compromise for balancing *T*_lum_ and Δ*T*_sol_ through porosity/thickness, positioning the aerogel-like samples among the most outstanding thermochromic films reported.^2,6,8,20,21,26,57–59^ and even comparable to moth-eye VO_2_ coatings, which require complex nanofabrication.^24^

The aerogel-like films also exhibit Δ*T*_IR_ values significantly higher than conventional VO_2_ films.^14,15^ For the 135 and 190 nm samples, the Δ*T*_IR_ values are 13% and 13.3%, respectively. However, with increasing thickness to 380 nm, Δ*T*_IR_ rises considerably to 29.8% and reaches a maximum of 35.5% for the 650 nm films. This remarkable infrared modulation further underscores the excellent performance of aerogel-like VO_2_ films, making them highly promising for smart window applications where both visible light and infrared modulation are crucial.

The previous results show how the high porosity of the VO_2_ aerogel-like films simultaneously enhances luminous transmittance and solar modulation. Figure S3 shows the refractive index of the aerogel-like films as a function of the thickness, with very low effective indices in the range 1.31–1.26. Thus, the highly open and low-density architecture (see Figure 5) minimizes reflection and visible absorption because the solid matrix is extremely thin and composed of very small VO_2_ crystals. The low refractive index contrast with air efficiently suppresses scattering (Figure 11a), an effect previously observed in antireflective aerogel-like TiO_2_ prepared by the same methodology.^28^ In parallel, the porous network increases the optical path in the near-infrared through multiple internal reflections, while the large surface area and abundant grain boundaries promote more abrupt and pronounced semiconductor-to-metal transitions, effectively boosting the optical contrast across the phase transition.^22^

The semiconductor-to-metal switching characteristics of the thin films were examined by recording the temperature-dependent optical transmittance during both the heating and cooling processes at λ = 2000 nm, as illustrated in the central panel of Figure 11. To calculate the transition temperature (*T*_t_), the derivative of the optical transmittance (*T*_r_, to distinguish from temperature *T*) with respect to temperature (*T*) was plotted against the temperature (*T*) for each heating and cooling branch, as shown in the bottom panel of Figure 11. This graphical representation of the derivative allows for identifying two inflection points indicating the phase transition in the optical properties of the films. Each curve was fitted to a Lorentzian function, as shown in the bottom panel of Figure 11. *T*_h_ and *T*_c_ are the transition temperatures for the heating and cooling cycles, while the average transition temperature (*T*_t_) is then calculated as *T*_t_ = (*T*_h_ + *T*_c_)/2.

The full width at half-maximum (FWHM) values (bottom panel in Figure 11) provide an insight into the sharpness of the transition, i.e., how abrupt the metal–insulator transition is in the thin film during the heating (FWHM_h_) and cooling (FWHM_c_) phases. The hysteresis width (Δ*T*_t_) is defined as the difference between *T*_h_ and *T*_c_, providing a measure of the hysteresis of the process. A wide hysteresis width indicates that the material can maintain its metallic or insulating phase over a broader range of temperatures during cooling or heating. In contrast, a smaller hysteresis width suggests greater symmetry in the transition.

Interpreting the transition temperatures and hysteresis loops of the aerogel-like films as a function of thickness is complex. The average transition temperature (Table 1) decreases with increasing thickness, from 59.4 °C for the 135 nm film to 58.5 °C and 53.2 °C for the 190 nm and 380 nm samples, respectively. This latter sample presents the minimum *T*_t_ for the studied samples, which is significantly lower than the *T*_t_ corresponding to crystalline undoped VO_2_ (*T*_t_ ∼ 68 °C).^51,56^ The transition hysteresis also decreases from Δ*T*_t_ = 14.2 °C to Δ*T*_t_ = 12.1 °C in the same thickness range (bottom panel in Figure 12a). For these samples, the reduced transition temperatures and the hysteresis evolution can be attributed to factors such as the distribution and size of VO_2_ crystals, the effect of the strain of the VO_2_ nanocrystal lattices observed in the HRTEM analysis, and the impact of the increasing porosity of the aerogel-like films with the thickness.^52,60^

**Figure 12:**
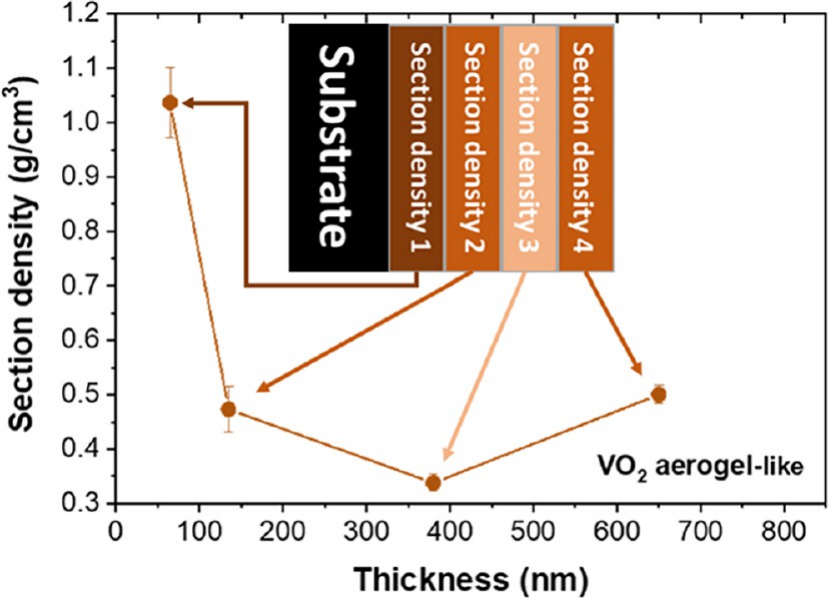
Evolution of the section density of the film as a function of the thickness for the VO_2_ aerogel-like films calculated using the data from Figure 1d.

However, for the 650 nm thick film, *T*_t_ increases to 66.4 °C, a value close to the VO_2_ nominal *T*_t_, and the transition hysteresis increases to 21.4 °C (Figure 11a). These results indicate that the VO_2_ crystal structure is relaxed in this sample. The evolution of the density of the aerogel-like VO_2_ films with thickness shows a density decrease with thickness up to 380 nm and then a slight increase for the film of 650 nm thick, giving rise to the plateau in Figure 1d. A similar plateau can be observed in the V_2_O_5_ precursor films (Figure 1c) and also in partly crystalline TiO_2_ films prepared by the same methodology.^28^ The shape of the curve in Figure 1d points out a thickness gradient density that decreases with the thickness. Plotting the section density of every cycle as shown in Figure 12, it can be noted that the minimum density is observed at 380 nm, and then it increases slightly for the 650 nm thick sample. This thickness increase is likely linked to the percolation of VO_2_ crystals, leading to the observed rise in *T*_t_. Thus, several authors have reported *T*_t_ close to the nominal value of VO_2_ in percolated nanoporous structures^57^ and in VO_2_ crystalline nanoparticles.^26,54^

Another notable feature in the optical transmittance curve of the 650 nm aerogel-like sample is the appearance of an absorption band around 1150 nm in the high-temperature spectrum (top panel of Figure 11a, highlighted with a vertical dashed line). This absorption corresponds to the formation in the films of a localized surface plasmon resonance (LSPR), which is not observed for lower thicknesses where light transmission decreases monotonically toward long wavelengths. Surface plasmon resonances in the range 1100–1200 nm have been reported for VO_2_ nanoparticles of different shapes and aspect ratios^26,54,61^ and in percolated nanoporous structures.^57^ Notably, VO_2_ localized surface plasmon resonances have been proposed as an effective tool to improve the solar regulation efficiency of VO_2_-based thermochromic systems, which can absorb infrared radiation without affecting visible light absorption.^26,51,57^

While the 380 nm aerogel-like films show a similar porous microstructure, they do not exhibit this localized absorption. Direct HRTEM evidence of nanocrystal size evolution is challenging to obtain because VO_2_ nanocrystals rapidly change under electron beam exposure; however, both the section density profile (Figure 12) and the refractive index evolution of the films (Figure S3) indicate an increase in surface density for the 650 nm sample. This densification very likely arises from partial aggregation and percolation of VO_2_ nanocrystals, enabling LSPR formation. Notably, the minimum section density occurs at 380 nm, after which the density increases with thickness, a trend also observed in the V_2_O_5_ precursor films (Figure 1c) and in previously reported aerogel-like nanocrystalline anatase films prepared by the same methodology once a critital thickness is reached.^28^ We hypothesize that LSPR in the thickest films is linked to the onset of nanocrystal percolation to give rise to larger structures with an associated increase in film density.

On the other hand, the thermochromic performance (Figure 11b and Table 1) of the RPAVD-O_2_ VO_2_ films is different from that of the aerogel-like samples. In these samples, the thermochromic operation is restricted to ultrathin films with thicknesses lower than 100 nm, similar to VO_2_ films prepared by magnetron sputtering, pulsed laser deposition, and other techniques.^60,62–64^ Thus, increasing the thickness, Δ*T*_sol_ rises by 4.1%, while *T*_lum_ decreases drastically by 20%. The Δ*T*_IR_ values are notably high for the two samples, reaching 21.6% for the thicker sample. The *T*_t_ decreases with the thickness from a value close to the nominal value of VO_2_ to 55.4 °C, a value significantly lower and in the range of the aerogel-like films with thicknesses lower than 380 nm. These findings are likely related to the transition from continuous percolated porous films to higher porosity films formed by more isolated nanograins observed in Figure 3b.

### 3.5 Encapsulation of Aerogel-like Films for Environmental Protection

VO_2_ is thermodynamically unstable and can be oxidized to V_2_O_5_ under air exposure over several months and accelerated under humid environments.^65^ This environmental instability limits the practical applicability of thermochromic VO_2_ films, nanoparticles, and nanocomposites, mainly for smart window applications.^66^ Currently, research is underway to synthesize hybrid materials that can almost entirely prevent the oxidation of VO_2_ without compromising its thermochromic properties.^6,8,66,67^ Thus, different solutions have been proposed to effectively delay the oxidation process, such as the use of inorganic protective coatings and shells and polymers.^6,67–69^

In this section, we present a preliminary study on the encapsulation of aerogel films using a high-performance polymer synthesized with the same reactor and technique used to synthesize the sacrificial vanadium polymer films. This encapsulating polymer is deposited by RPAVD of adamantane (hereafter ADA films), forming a compact and conformal layer on top of the aerogel-like VO_2_ film (see Figure S4). These ADA films have been applied to encapsulate hybrid halide perovskite cells, enabling their use in high humidity conditions and underwater operation^70^ and MoS_2_ 2D layers for strain engineering.^33^ The ADA films are fully transparent in the visible and IR regions with a refractive index of 1.57 at 550 nm. Besides, the films are insoluble, thermally stable, and robust against harsh chemical environments.^31^ Another important aspect is the possibility of providing mechanical stability to the aerogel-like film surfaces thanks to the relatively high hardness and high Young’s modulus values of the films (7.5–10.0 GPa).^33^ Note that this aspect is crucial for using aerogel-like films in smart-coating applications.

Figure 13 shows the spectral transmittance of a 375 nm aerogel-like film before (purple line) and after (red line) the encapsulation with a 150 nm thick ADA film (both at RT and 90 °C). In addition, the ADA encapsulated sample has been measured after a long period of exposure to atmospheric conditions (humidity values between 35 and 50% for 550 days). The transmittance of the aerogel-like thin film right after the encapsulation with the adamantane plasma polymer remains essentially unchanged (Figure 13a). Only minimal changes in the NIR of the ADA-encapsulated film transmittance are appreciated, of less than 5% compared to the original film, likely due to the antireflectance effects of the adamantane film conformal coating. However, the thermochromic parameters are affected. As shown in Figure 13b, the hysteresis of the ADA-VO_2_ film retains essentially the same shape and symmetry but is shifted to higher temperatures, with the corresponding parameters summarized in Table 2. Encapsulation with the adamantane coating increases the transition temperature by 12.9 °C compared to the original film (from 53.2 °C to 66.1 °C). The rigid ADA polymer provides mechanical stabilization to the highly porous VO_2_ aerogel-like films; by conformally clamping the nanocrystalline network (see Figure S4), it redistributes local stresses and promotes partial relaxation of internal strain in the VO_2_ nanocrystals. This strain relaxation likely contributes to the observed shift in transition temperature after encapsulation. Meanwhile, the luminous transmittance remains nearly unchanged, decreasing slightly from 58.6 % to 56.5 %, and ΔTsol increases from 14.7 % to 16.5 %.

**Figure 13:**
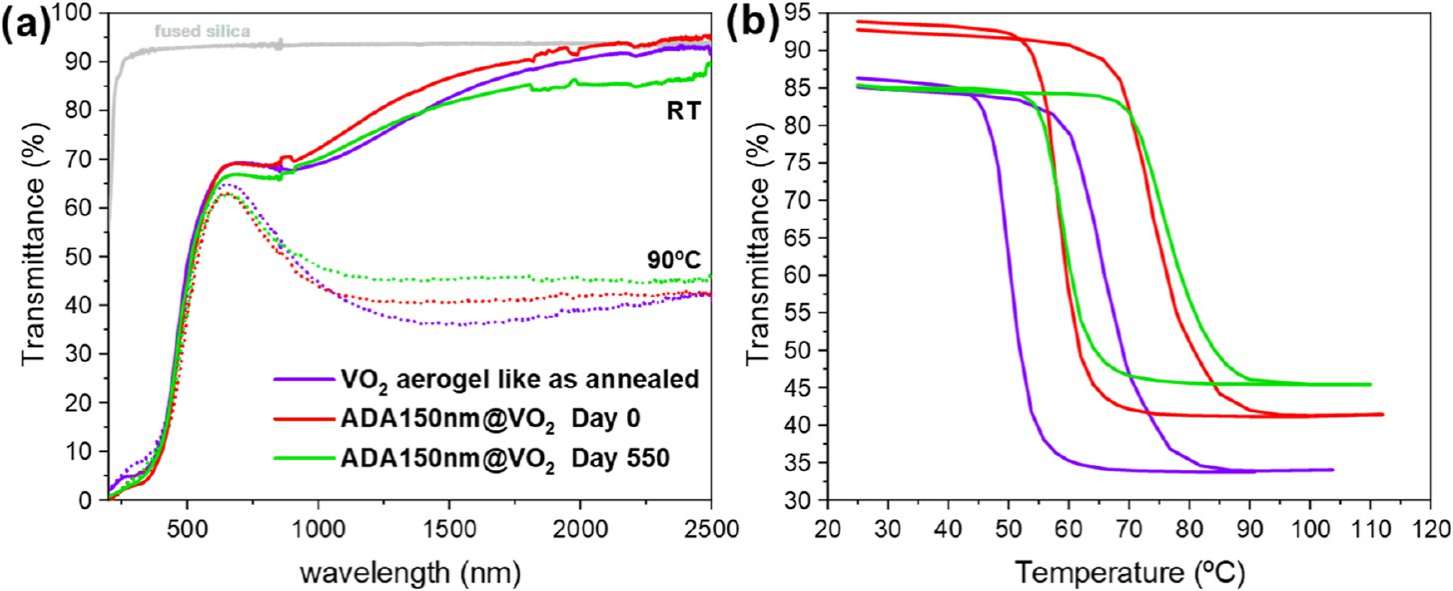
Effects of 150 nm adamantane plasma polymer encapsulation on a 380 nm aerogel-like VO_2_. (a) Temperature-dependent optical transmittance spectra in the UV–vis–NIR region at room temperature (solid lines) and 90 °C (dotted lines). (b) Hysteresis loops corresponding to the films in (a).

**Table 2:** Optical Parameters of VO_2_ Aerogel-like Sample Coated with 150 nm of RPAVD-ADA Coating as Deposited and after 550 Days of Ambient Exposure

		*T*_lum_ (%)			*T*_sol_ (%)			*T*_IR_ (%)		
sample	*T*_t_ (°C)	low *T*	high *T*	Δ*T*_lum_	low *T*	high *T*	Δ*T*_sol_	low *T*	high *T*	Δ*T*_IR_
VO_2_ aerogel as annealed	53.2	58.6	55.8	2.8	61.2	46.5	14.7	74.3	44.5	29.8
ADA/VO_2_ day 0	66.1	56.5	52.4	4.1	61.4	44.8	16.5	77.2	45.0	32.2
ADA/VO_2_ day 550	67.8	56.5	53.6	2.9	59.7	47.1	12.6	73.6	48.6	25.0

The encapsulating effect of the adamantane film becomes evident in the transmittance spectra of the hot and cold states after 550 days of air exposure. In this case, the Δ*T*_IR_ compared to the measurements taken on day 0 decreases only by 7.2%, whereas the hysteresis of the process remains practically unchanged, and *T*_t_ increases very slightly by 1.7 °C. In addition, Δ*T*_sol_, decreases by 4.0% (from 16.5 to 12.6%), while *T*_lum_ remains almost unaffected. In contrast, an unprotected aerogel-like VO_2_ film undergoes complete oxidation, reverting to an optical transmittance similar to that of the precursor aerogel-like V_2_O_5_ film (Figure S5).

The above results show the potential of the remote plasma polymerization technique to encapsulate aerogel-like VO_2_ films and significantly increase their environmental and mechanical stabilities. This study is positive but preliminary. A more comprehensive analysis is needed to optimize parameters such as the optimal thickness of the polymeric layer, degree of cross-linking as a function of the preparation conditions, and conformality of the polymer to the internal porous structure. Systematic characterization of the encapsulated VO_2_ films as a function of temperature and degree of humidity is also needed. These studies are currently underway.

## 4 Conclusions

In this work, we present a low-temperature methodology for the synthesis of VO_2_ aerogel-type thermochromic films based on a sequential remote plasma deposition and plasma processing steps followed by a reductive annealing treatment. The results confirm the generality of the synthetic methodology and its potential to develop ultraporous oxide films with a wide range of compositions. In this procedure, high porosity is achieved by remote plasma polymerization of a high C to V ratio precursor, such as vanadyl phthalocyanine with C/V = 32, and subsequent plasma etching removal of the organic part of the film (C and N species) by formation of volatile compounds which are pumped out. Simultaneously, oxidation of the V^4+^ cation occurs, giving rise to partially crystalline V_2_O_5_ films. Utilizing a cyclic process of polymerization and oxidation, we have been able to control the thickness and porous structure of the material.

After annealing in an Ar atmosphere, we obtained ultraporous films composed of nanocrystalline monoclinic VO_2_, free of amorphous regions or secondary crystalline phases, preserving the interconnected aerogel-like morphology of the V_5_O_5_ films. These VO_2_ aerogel films exhibit thermochromic behavior, with luminous transmittance ranging from 79.6% to 54.4% and solar modulation values of 14.7% and 18.8% for 380 nm and 650 nm films, respectively. The thicker films show a surface plasmon resonance near 1150 nm at temperatures above Tt, attributed to the percolation of the nanocrystalline network, which enhances their thermochromic modulation efficiency.

We have fabricated more compact V_2_O_5_ films using the same precursor and an oxygen plasma RPAVD process in a single synthetic step. After applying the same reductive annealing used for the aerogel films, we obtained crystalline thermochromic VO_2_ films with light transmittance above 50% and solar modulation up to 10.3% for thicknesses below 100 nm. The results prove the versatility of the synthetic approach that can be applied to synthesizing compact and ultraporous VO_2_ films from the same precursor by changing the synthesis conditions.

The main goal of this work is to demonstrate the potential of plasma processes to produce aerogel-like thermochromic VO_2_ films. The methodology is inherently compatible with conformal and on-device deposition,^28^ elemental doping (e.g., W, Mg) and multilayer optical designs commonly used to enhance VO_2_ transparency and thermochromic performance for smart solar modulation. In addition, the same strategy provides an effective route for encapsulating ultraporous films with transparent, robust, and environmentally stable coatings, while maintaining the industrial scalability and eco-friendly characteristics of plasma-based processes.

## Supplementary Material



## Notes

Additional optical, Raman, SEM, and XPS characterizations of selected VOPC-RPAVD and aerogel-like VO_2_ films (PDF)
