# Supplementary material for: Enhanced Luminous Transmission and Solar Modulation in Thermochromic VO2 Aerogel-like Films via Remote Plasma Deposition
Source: ACS Appl Mater Interfaces. 2025 Sep 22;17(39):55172–88. doi: 10.1021/acsami.5c07264 (PMC12492333; doi:10.1021/acsami.5c07264)
Supplement: Supplementary file 1 [file am5c07264_si_001.pdf]

Supporting Information

Enhanced Luminous Transmission and Solar Modulation in Thermochromic VO_2 Aerogel-Like Films via Remote Plasma Deposition

Jose Manuel Obrero,[a] Gloria Patricia Moreno-Martinez,[a] Teresa Cristina Rojas,[b] Francisco Javier Ferrer,[c,d] Francisco G. Moscoso,[e] Lidia Contreras-Bernal,[a,f] Javier Castillo-Seoane,[a] Fernando Nuñez-Galvez,[a] Francisco Javier Aparicio Rebollo,[a] Ana Borras,[a] Juan Ramon Sanchez-Valencia,[a] Angel Barranco[a*]*

a) Nanotechnology on Surfaces and Plasma Laboratory, Materials Science Institute of Seville (CSIC-US), C/ Américo Vespucio 49, 41092, Seville, Spain.

b) Tribology and Surface Protection Group. Materials Science Institute of Seville (CSIC-US), C/ Américo Vespucio 49, 41092, Seville, Spain.

c) Centro Nacional de Aceleradores (Universidad de Sevilla, CSIC, Junta de Andalucía). Avda. Tomas Alba Edison 7, 4092, Sevilla

d) Departamento de Física Atómica, Molecular y Nuclear, Universidad de Sevilla, Aptdo 1065, 41012 Sevilla, Spain

e) Center for Nanoscience and Sustainable Technologies (CNATS), Departamento de Sistemas Físicos, Químicos y Naturales, Universidad Pablo de Olavide, Ctra. Utrera km. 1, Sevilla 41013, Spain.

f) Departamento de Ingeniería y Ciencia de los Materiales y el Transporte, EPS-Universidad de Sevilla, c/Virgen de África 7, 41011, Sevilla, Spain.

Corresponding author e-mail: angel.barranco@csic.es; jrsanchez@icmse.csic.es

S1. UV-Vis spectra and FESEM micrographs of RPAVD-Ar polymer films

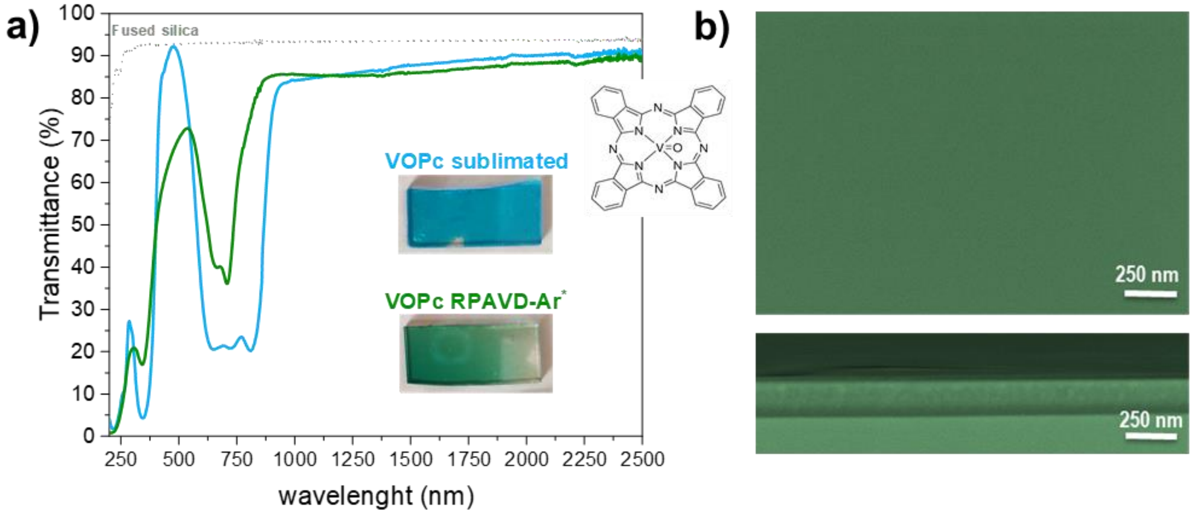

Figure S1. (a) UV-VIS-NIR Transmittance spectra of a plasma polymerized VOPc film by RPAVD-Ar and a reference sublimated VOPc films. It also includes photographs of the films deposited on fused silica. (b) FESEM normal and cross-sectional micrographs of the RPAVD-Ar VOPc layer used as a sacrificial layer to synthesize aerogel-like V_2O_5 and VO_2 films.

S2. Raman analysis of aerogel-like V_2O_5 and VO_2 thin films

The vanadium oxide films were characterized by room temperature Raman before and after annealing and analyzed according to the literature.[1-5] As depicted in **Figure 9c)**, the as-deposited aerogel-like thin film exhibits characteristic peaks indicative of pure crystalline α-V_2O_5, congruent with the previous XRD and TEM characterizations, with no observable traces of other vanadium oxides or compounds

The as-deposited V_2O_5 aerogel-like thin film exhibits characteristic peaks indicative of pure crystalline α-V_2O_5, congruent with the previous XRD and TEM characterizations, with no observable traces of other vanadium oxides or compounds. The structural analysis reveals 21 Raman modes ($7A_g + 3B_{1g} + 7B_{2g} + 4B_{3g}$). The structural analysis reveals 21 Raman modes ($7A_g + 3B_{1g} + 7B_{2g} + 4B_{3g}$). Notably, the high-frequency Raman peak observed at 992 cm^{-1} (A_g) corresponds to the terminal oxygen V=O stretching mode, resulting from unshared oxygen. Another significant peak at 702 cm^{-1} (B_{1g}/ B_{3g}) is attributed to the asymmetric stretching mode of doubly coordinated oxygen in V-$O_{(2)}$-V, arising from oxygen shared at the corners common to two pyramids. Unfortunately, the 528 cm^{-1} (A_g) and 507 cm^{-1} peaks overlap with the silicon substrate peak. These peaks are associated with the triply coordinated oxygen (V-$O_{(3)}$-V) stretching mode, resulting from oxygens shared at the edges common to the three pyramids. Further insights into the vibrational characteristics include the identification of peaks at 483 (A_g) and 304 cm^{-1} (A_g), corresponding to the bending vibrations of bridging V-O-V (doubly coordinated oxygen) and triply coordinated oxygen (V-$O_{(3)}$-V) bonds, respectively. Additionally, the peaks at 405 (A_g) and 284 cm^{-1} (B_{1g}/ B_{3g}) are associated with the bending vibration of V=O bonds. Two low-frequency Raman peaks, discernible at 198 (A_g/ B_{2g}) and 150 cm^{-1} (B_{1g}/ B_{3g}), are indicative of lattice vibrations and provide insights into the vibrational modes associated with the layered structure and

the structural features of α-V_2O_5.[1,6-8] The Raman spectrum for the aerogel-like annealed sample displays all the peaks attributed to VO_2 (M1), consistent with previously reported data.[1,2] According to group theory, the M1 phase of VO_2 possesses 18 Raman active modes ($9A_g$ and $9B_g$), with 12 modes observed in the VO_2 thin film at ∼138, 194, 223, 262, 307, 338, 387, 439, 442, 497, 583, and 613 cm^{-1} (**Figure 9c**). Importantly, no other phases or polymorphs were detected. The slight discrepancies between our data and those in the literature refer to small shifts in the Raman peak positions (in cm^{-1}). These shifts can be attributed to the pronounced difference in thermal expansion coefficients between VO_2 and the silicon substrate, which induces heightened internal strain in films deposited on this substrate, thus explaining the observed variations.[3] The low-frequency phonons at 194 and 223 cm^{-1} are associated with lattice motion involving V-V bonds, while the remaining peaks correspond to vibrational modes of V-O bonds. The bands in the low wavenumber region (< 400 cm^{-1}) are attributed to V−O−V bending modes. As the wavenumber transitions to the intermediate range (400−800 cm^{-1}), the bands correspond to V−O−V stretching modes. The broad peak at 613 cm^{-1} is a convolution of the 588, 613, and 661 cm^{-1} peaks. The bands in the high wavenumber range (> 800 cm^{-1}) can be assigned to V=O stretching modes indicative of distorted octahedra and square pyramids.

References:

[1] Basu, R.; Prasad, A. K.; Dhara, S.; Das, A. Role of Vanadyl Oxygen in Understanding Metallic Behavior of V2O5(001) Nanorods. *J. Phys. Chem. C* **2016**, *120* (46), 26539–26543.

[2] Chen, X.-B. Assignment of the Raman Modes of VO2 in the Monoclinic Insulating Phase. *J. Korean Phys. Soc.* **2011**, *58* (1), 100–104.

[3] Ureña-Begara, F.; Crunteanu, A.; Raskin, J.-P. Raman and XPS Characterization of Vanadium Oxide Thin Films with Temperature. *Appl. Surf. Sci.* **2017**, *403*, 717–727.

[4] Silversmit, G.; Depla, D.; Poelman, H.; Marin, G. B.; Gryse, R. D. Determination of the V2p XPS Binding Energies for Different Vanadium Oxidation States (V5+ to V0+). *J. Electron Spectrosc. Relat. Phenom.* **2004**, *135* (2), 167–175.

[5] Sun, Y.; Jiang, S.; Bi, W.; Long, R.; Tan, X.; Wu, C.; Wei, S.; Xie, Y. New Aspects of Size-Dependent Metal-Insulator Transition in Synthetic Single-Domain Monoclinic Vanadium Dioxide Nanocrystals. *Nanoscale* **2011**, *3* (10), 4394–4401.

[6] Lee, S.-H.; Cheong, H. M.; Je Seong, M.; Liu, P.; Tracy, C. E.; Mascarenhas, A.; Pitts, J. R.; Deb, S. K. Microstructure Study of Amorphous Vanadium Oxide Thin Films Using Raman Spectroscopy. *J. Appl. Phys.* **2002**, *92* (4), 1893–1897.

[7] Lee, S.-H.; Cheong, H. M.; Seong, M. J.; Liu, P.; Tracy, C. E.; Mascarenhas, A.; Pitts, J. R.; Deb, S. K. Raman Spectroscopic Studies of Amorphous Vanadium Oxide Thin Films. *Solid State Ion.* **2003**, *165* (1), 111–116.

[8] Shvets, P.; Dikaya, O.; Maksimova, K.; Goikhman, A. A Review of Raman Spectroscopy of Vanadium Oxides. *J. Raman Spectrosc.* **2019**, *50* (8), 1226–1244.

S3. XPS characterisation of sublimated and RPAVD-Ar VOPc films.

The characterization and assignment of the XPS peaks were carried out in accordance with procedures and reference data reported in the literature, including the evaluation of peak positions, chemical states, and the fitting of the spectra using Gaussian–Lorentzian profiles.[1-4]

a) XPS characterization of VOPc plasma polymers.

The sublimated and VOPc plasma polymeric films deposited by RPAVD-Ar were analysed by XPS. The survey spectra and the atomic/relative percentages of each element (V, C, O, and N) are presented in **Figures S2a)** and **S2b)**, respectively. Significant differences were observed in the relative amounts of N, C, and O obtained from XPS analysis with respect to V (**Figure S2b**). Specifically, $(N/V)_{XPS}$=5.0, $(C/V)_{XPS}$=27.0, and $(O/V)_{XPS}$=6.5, compared to the sublimated film, where $(N/V)_{sublimated}$=7.0, $(C/V)_{sublimated}$=31.0, and (O/V)sublimated=1.0, values that closely approximate those of the empirical formula of VOPc ($C_{32}H_{16}N_8OV$). Polymerisation via RPAVD-Ar resulted in a slight decrease in C and N content, accompanied by an increase in oxygen content, which, in turn, led to the partial oxidation of the VOPc molecule and the generation of volatile carbon and nitrogen products. This oxygen enrichment is a common phenomenon in RPAVD techniques, attributed to post-deposition reactions with air and/or direct incorporation of residual oxygen from the reactor during deposition.[5,6]

To observe the changes undergone by the molecule during polymerization via RPAVD, XPS zone spectroscopy measurements were performed in the O1s+V2p, C1s, and N1s regions, and are shown in **Figures S2c), S2d) and S2e)**, respectively, for the sublimated and polymerized VOPc films. According to the O1s+V2p zone spectra (**Figure S2c)**), the oxidation state of vanadium in both cases can be estimated from the position of the $V2p_{3/2}$ peak. In the case of the sublimated VOPc film, a single intense peak at 416.4 eV is observed, confirming the exclusive presence of

vanadium in its V^{4+} form. However, when the molecule undergoes plasma polymerization, the residual oxygen in the chamber oxidizes some of the V^{4+} to V^{5+}, as evidenced by the appearance of a second peak at 517.6 eV. Approximately 25% of the total V content oxidizes to the highest oxidation state during this process. The C1s spectra for the same samples are shown in **Figure S2d)**. Both exhibit C1s species characteristic of phthalocyanines: one corresponding to saturated C-H carbons, situated at a binding energy of 285.0 eV, and another in the unsaturated C=C carbon region at 284.5 eV. A second peak of C-N at 286.5 eV, associated with the carbon bonded to the pyridinic nitrogen of phthalocyanine ($N_β$), and a third peak of C=N at 288.0 eV, related to the carbon bonded to the pyrrolic nitrogen ($N_α$), are highlighted. Additionally, a minor peak at 291.0 eV corresponds to the π-π^* interaction of the phthalocyanine rings.

The incorporation of residual oxygen into the polymerized film is manifested by an increase in the peak at 288.0 eV, from 8.3% of the total C in the sublimated films to 15.2% in the polymerized films, where C=O bands are also observed. Thus, it is evident that some of the carbons located in the isoindole groups are partially oxidized. This phenomenon is more pronounced in the N1s spectra of **Figure S2e)**. Thus, in the sublimated film, only $N_β$ and $N_α$ peaks at 398.8 and 399.3 eV, respectively, are observed in the same proportion. However, 80% of the surface Nα peaks are partially fragmented during the plasma polymerization, generating more oxidized species, as indicated by a 400.2 eV peak assigned to the -N-(C=O)-O- species.

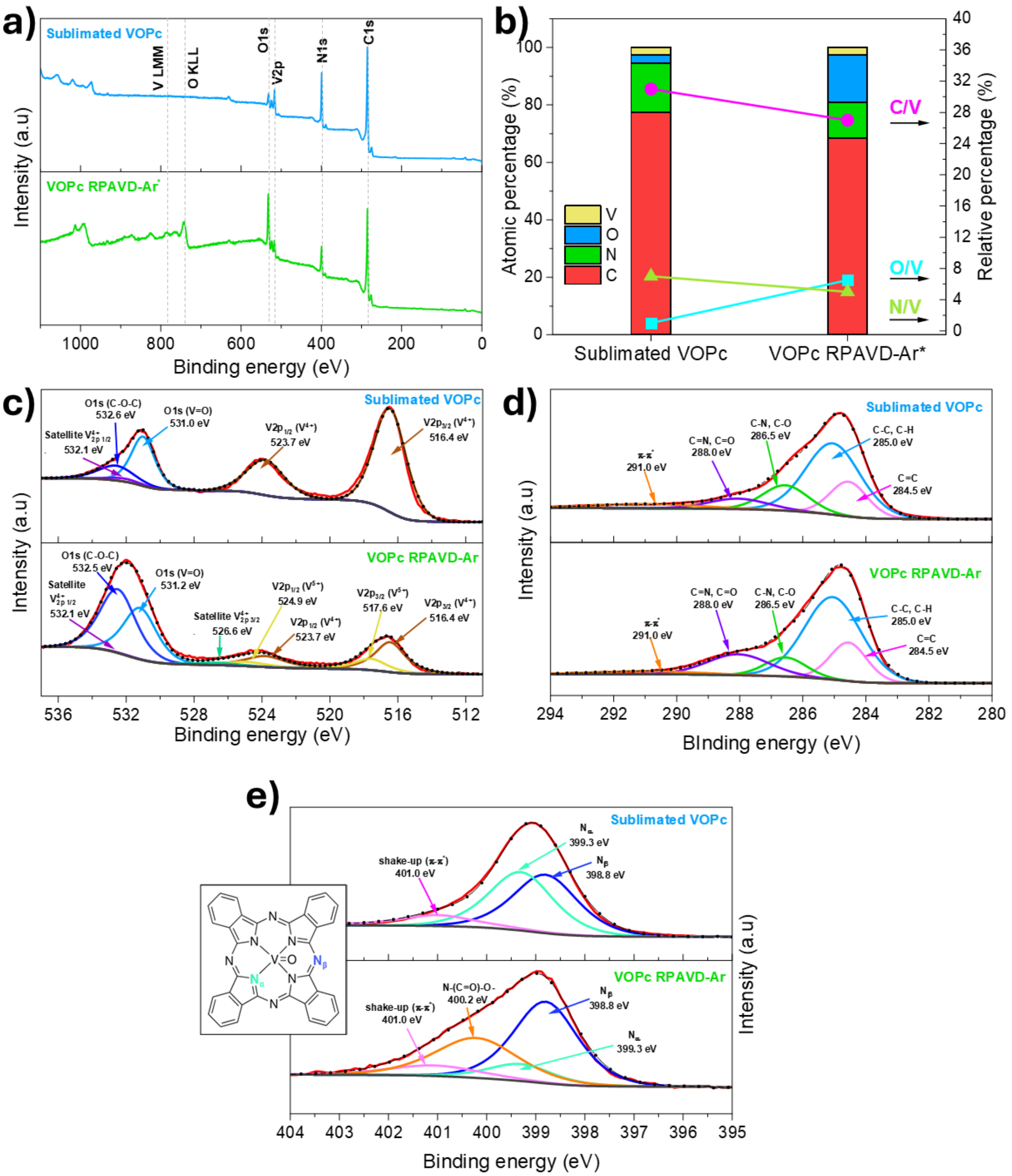

Figure S2. (a) XPS survey spectra of a reference VOPc sublimated films and a VOPc plasma polymer deposited by RPAVD-Ar. (b) Calculated surface atomic percentages. XPS core-level spectra of the films, corresponding to (c) V2p + O1s, (d) C1s, and (e) N1s regions

b) Supplementary information regarding the XPS characterization of V_2O_5 and VO_2 thin films.

Table S1. XPS spectral fitting parameters for V2p species, showing main and satellite peaks for aerogel-like and RPAVD-O_2 films (as-deposited and annealed). ΔE_{sat} represents the binding energy difference between each V2p satellite peak and its corresponding main peak, while I_{sat}/I_{main} denotes the intensity ratio of the satellite to the main peak. Peak position from Reference [7] are indicated in parentheses.

			**Main peak**		**Satellite peak**	
			**BE (eV)**	**FWHM (eV)**	**ΔE_{sat} (eV)**	**I_{sat}/I_{main}**
**Aerogel-like**	**As deposited**	V^{5+} $2p_{3/2}$	517.58 (517.26)	2.1	14.7 (13.7)	0.06 (0.10)
		V^{5+} $2p_{1/2}$	V^{5+} $2p_{3/2}$ + 7.36 (V^{5+} $2p_{3/2}$ + 7.33)	3.5	-	-
	**Annealed**	V^{5+} $2p_{3/2}$	517.58 (517.26)	2.2	14.7 (13.7)	0.06 (0.10)
		V^{5+} $2p_{1/2}$	V^{5+} $2p_{3/2}$ + 7.36 (V^{5+} $2p_{3/2}$ + 7.33)	3.5	-	-
		V^{4+} $2p_{3/2}$	516.40 (516.04)	2.6	10.2 (11.8)	0.11 (0.10)
		V^{4+} $2p_{1/2}$	V^{4+} $2p_{3/2}$ + 7.36 (V^{4+} $2p_{3/2}$ + 7.33)	3.2	8.3 (7.9)	0.12 (0.30)
**RPAVD-O_2**	**As deposited**	V^{5+} $2p_{3/2}$	517.63 (517.26)	1.8	14.7 (13.7)	0.06 (0.10)
		V^{5+} $2p_{1/2}$	V^{5+} $2p_{3/2}$ + 7.36 (V^{5+} $2p_{3/2}$ + 7.33)	3.4	-	-
	**Annealed**	V^{5+} $2p_{3/2}$	517.70 (517.26)	2.3	14.7 (13.7)	0.06 (0.10)
		V^{5+} $2p_{1/2}$	V^{5+} $2p_{3/2}$ + 7.36 (V^{5+} $2p_{3/2}$ + 7.33)	3.8	-	-
		V^{4+} $2p_{3/2}$	516.40 (516.04)	2.2	10.2 (11.8)	0.11 (0.10)
		V^{4+} $2p_{1/2}$	V^{4+} $2p_{3/2}$ + 7.36 (V^{4+} $2p_{3/2}$ + 7.33)	2.9	8.3 (7.9)	0.12 (0.30)

Table S2. XPS fitting parameters for the V^{5+} $2p_{3/2}$, V^{4+} $2p_{3/2}$ and O1s (oxide) peaks for vanadium oxide standards (experimental data) and reference values reported in the literature.

**BE V^{5+} $2p_{3/2}$ (eV)**	**BE V^{4+} $V2p_{3/2}$ (eV)**	**BE O1s oxide (eV)**	**Reference**
517.2	515.9	530.0	[7]
517.9	516.4	530.3	[1]
517.5	516.4	530.8	[8]
517.0	515.5	530.0	[9]
517.2	516.1	530.5	[10]
517.7	517.1	-	[11]
517.5	516.2	530.0	[12]
517.3	516.0	529.3	[13]
517.0	516.4	530.0	[14]
**517.6**	**516.4**	**530.0**	**This work**

References

[1] Biesinger, M. C.; Lau, L. W. M.; Gerson, A. R.; Smart, R. S. C. Resolving Surface Chemical States in XPS Analysis of First Row Transition Metals, Oxides and Hydroxides: Sc, Ti, V, Cu and Zn. Appl. Surf. Sci. **2010**, 257 (3), 887–898.

[2] Moulder, J. F.; Chastain, J. Handbook of X-Ray Photoelectron Spectroscopy: A Reference Book of Standard Spectra for Identification and Interpretation of XPS Data; Physical Electronics Division, Perkin-Elmer Corp.: Eden Prairie, Minn., **1992**.

[3] Beamson, G.; Briggs, D. High Resolution XPS of Organic Polymers: The Scienta ESCA300 Database; Wiley, **1992**.

[4] Biesinger, M. C. Accessing the Robustness of Adventitious Carbon for Charge Referencing (Correction) Purposes in XPS Analysis: Insights from a Multi-User Facility Data Review. Appl. Surf. Sci. **2022**, 597, 153681.

[5] A. Barranco, M. Bielmann, R. Widmer, P. Groening, Plasma polymerization of Rhodamine 6G thin films, Adv. Eng. Mater. 2005, 7, 396.
[6] H. Yasuda, Plasma Polymerization, Academic Press, 1985.
[7] Silversmit, G.; Depla, D.; Poelman, H.; Marin, G. B.; Gryse, R. D. Determination of the V2p XPS Binding Energies for Different Vanadium Oxidation States (V5+ to V0+). J. Electron Spectrosc. Relat. Phenom. 2004, 135 (2), 167–175. https://doi.org/10.1016/j.elspec.2004.03.004.
[8] Prokeš, J.; Košutová, T.; Kousal, J.; Kuzminova, A.; Kylián, O. Novel Technique to Produce Porous Thermochromic VO2 Nanoparticle Films Using Gas Aggregation Source. Scientific Reports 2025, 15 (1), 1755. https://doi.org/10.1038/s41598-025-86272-9.
[9] Zhu, M.; Zhang, D.; Yu, H. Tailoring the Thermochromic Properties of Sputter-Deposited VO2 Films by O2+ Ion Irradiation. Infrared Physics & Technology 2023, 133, 104844. https://doi.org/10.1016/j.infrared.2023.104844.
[10] Basso, M.; Colusso, E.; Carraro, C.; Kalha, C.; Riaz, A. A.; Bombardelli, G.; Napolitani, E.; Chen, Y.; Jasieniak, J.; Ratcliff, L. E.; Thakur, P. K.; Lee, T.-L.; Regoutz, A.; Martucci, A. Rapid Laser-Induced Low Temperature Crystallization of Thermochromic VO2 Sol-Gel Thin Films. Applied Surface Science 2023, 631, 157507. https://doi.org/10.1016/j.apsusc.2023.157507.
[11] Zhang, Z.; Guo, M.; Tang, Y.; Liu, C.; Zhou, J.; Yuan, J.; Gu, J. High Areal Capacitance of Vanadium Oxides Intercalated Ti3C2 MXene for Flexible Supercapacitors with High Mass Loading. Nanotechnology 2020, 31 (16), 165403. https://doi.org/10.1088/1361-6528/ab6689.
[12] Wu, J.; Wang, Z.; Li, B.; Liu, B.; Zhao, X.; Tang, G.; Zeng, D.; Tian, S. Facile Synthesis of Island-like ZrO2-VO2 Composite Films with Enhanced Thermochromic Performance for Smart Windows. Materials 2023, 16 (1). https://doi.org/10.3390/ma16010273.
[13] Wang, Z.; Li, B.; Tian, S.; Liu, B.; Zhao, X.; Zhou, X.; Tang, G.; Pang, A. Acid Solution Processed VO2-Based Composite Films with Enhanced Thermochromic Properties for Smart Windows. Materials 2021, 14 (17). https://doi.org/10.3390/ma14174927.
[14] Gracia, F.; Yubero, F.; Espinós, J. P.; González-Elipe, A. R. First Nucleation Steps of Vanadium Oxide Thin Films Studied by XPS Inelastic Peak Shape Analysis. Applied Surface Science 2005, 252 (1), 189–195. https://doi.org/10.1016/j.apsusc.2005.01.115.

S4. Refractive index evolution vs thickness for VO_2 aerogel-like films.

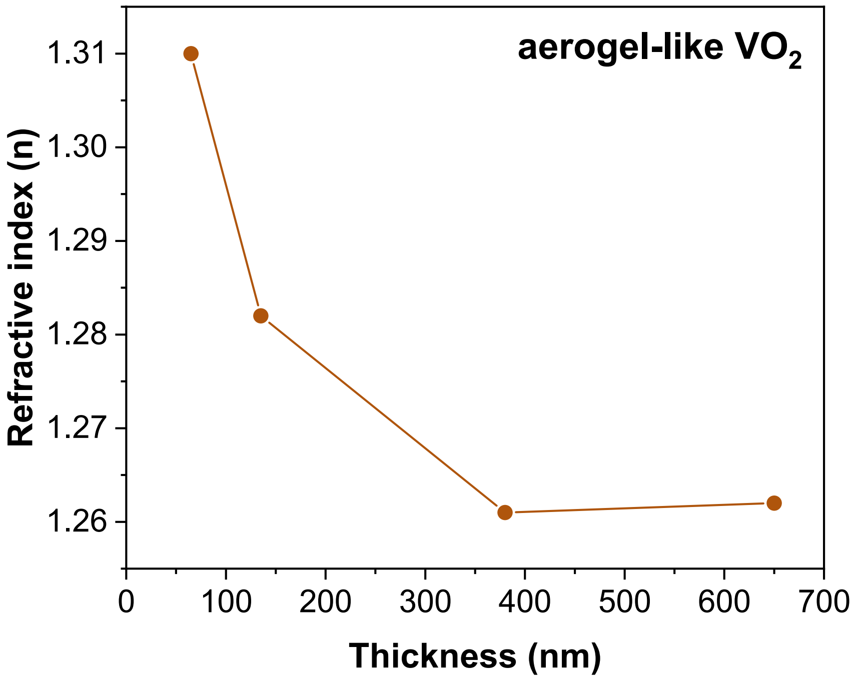

Figure S3. Refractive index determined in the IR region for VO_2 aerogel-like films by variable angle spectroscopic ellipsometry (VASE).

S5. Additional analysis of ADA encapsulated VO_2 thin films for environmental protection.

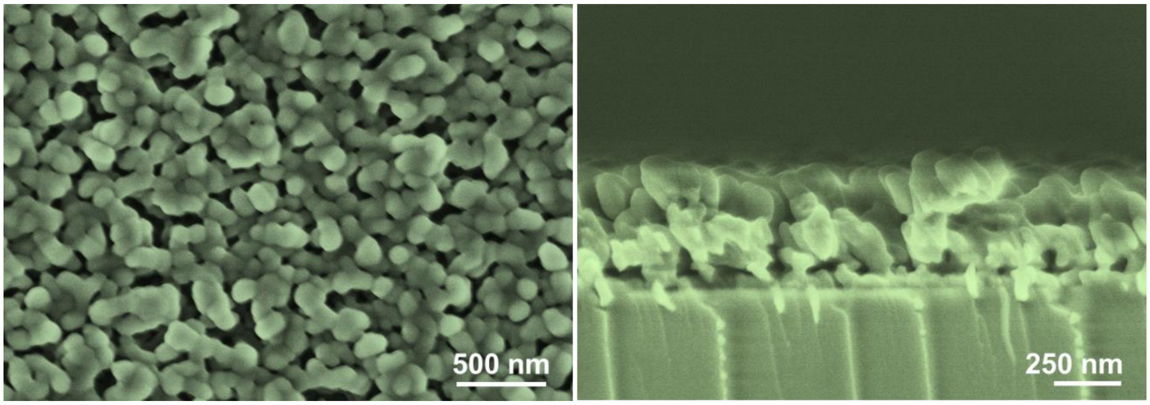

Figure S4. SEM top-view (a) and cross-sectional (b) micrographs of a VO_2 aerogel-like film encapsulated with adamantane plasma polymer.

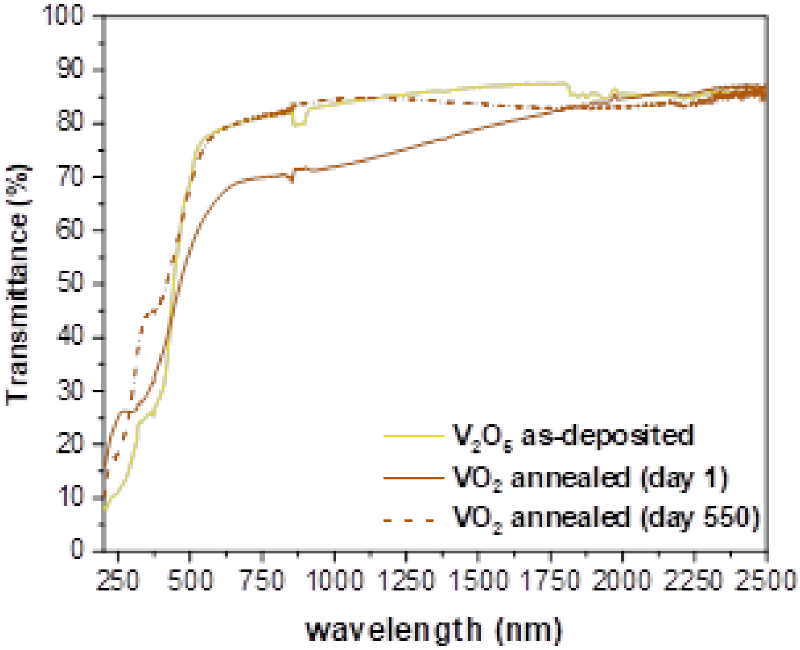

Figure S5. Optical transmittance spectra of the as-deposited V_2O_5 thin film, the VO_2 thin film after annealing (day 1), and the same VO_2 thin film after 550 days of exposure to ambient conditions.
